# Flexibility of Oral Cholera Vaccine Dosing—A Randomized Controlled Trial Measuring Immune Responses Following Alternative Vaccination Schedules in a Cholera Hyper-Endemic Zone

**DOI:** 10.1371/journal.pntd.0003574

**Published:** 2015-03-12

**Authors:** Suman Kanungo, Sachin N. Desai, Ranjan Kumar Nandy, Mihir Kumar Bhattacharya, Deok Ryun Kim, Anuradha Sinha, Tanmay Mahapatra, Jae Seung Yang, Anna Lena Lopez, Byomkesh Manna, Barnali Bannerjee, Mohammad Ali, Mandeep Singh Dhingra, Ananga Mohan Chandra, John D. Clemens, Dipika Sur, Thomas F. Wierzba

**Affiliations:** 1 Division of Epidemiology, National Institute of Cholera and Enteric Diseases, Kolkata, India; 2 Development and Delivery Unit, International Vaccine Institute, Seoul, Republic of Korea; 3 Division of Bacteriology, National Institute of Cholera and Enteric Diseases, Kolkata, India; 4 Division of Clinical Medicine, National Institute of Cholera and Enteric Diseases, Kolkata, India; 5 Laboratory Science Division, International Vaccine Institute, Seoul, Republic of Korea; 6 University of the Philippines Manila, National Institutes of Health, Manila, Philippines; 7 Division of Data Management, National Institute of Cholera and Enteric Diseases, Kolkata, India; 8 Department of International Health, Johns Hopkins Bloomberg School of Public Health, Baltimore, United States of America; 9 Clinical Research and Development, Shantha Biotechnics Private Limited, Hyderabad, India; 10 Department of Physiology, Calcutta University, Kolkata; 11 Office of the Executive Director, International Centre for Diarrheal Disease Research, Bangladesh; 12 Department of Epidemiology, University of California Los Angeles Fielding School of Public Health, Los Angeles, United States of America; 13 Office of the Scientific Director, PATH India Office, New Delhi, India; Massachusetts General Hospital, UNITED STATES

## Abstract

**Background:**

A bivalent killed whole cell oral cholera vaccine has been found to be safe and efficacious for five years in the cholera endemic setting of Kolkata, India, when given in a two dose schedule, two weeks apart. A randomized controlled trial revealed that the immune response was not significantly increased following the second dose compared to that after the first dose. We aimed to evaluate the impact of an extended four week dosing schedule on vibriocidal response.

**Methodology/Principal Findings:**

In this double blind randomized controlled non-inferiority trial, 356 Indian, non-pregnant residents aged 1 year or older were randomized to receive two doses of oral cholera vaccine at 14 and 28 day intervals. We compared vibriocidal immune responses between these schedules. Among adults, no significant differences were noted when comparing the rates of seroconversion for *V*. *cholerae O1 Inaba* following two dose regimens administered at a 14 day interval (55%) vs the 28 day interval (58%). Similarly, no differences in seroconversion were demonstrated in children comparing the 14 (80%) and 28 day intervals (77%). Following 14 and 28 day dosing intervals, vibriocidal response rates against *V*. *cholerae* O1 Ogawa were 45% and 49% in adults and 73% and 72% in children respectively. Responses were lower for *V*. *cholerae* O139, but similar between dosing schedules for adults (20%, 20%) and children (28%, 20%).

**Conclusions/Significance:**

Comparable immune responses and safety profiles between the two dosing schedules support the option for increased flexibility of current OCV dosing. Further operational research using a longer dosing regimen will provide answers to improve implementation and delivery of cholera vaccination in endemic and epidemic outbreak scenarios.

## Author Summary

The five year efficacy results of the bivalent, killed whole cell oral cholera vaccine was shown to offer 65% protection in cholera endemic Kolkata. Currently, two oral cholera vaccines (OCV) are prequalified by the World Health Organization: the whole cell recombinant cholera toxin B subunit vaccine (Dukoral), and the bivalent killed whole cell only OCV (Shanchol). Shanchol, which is less expensive and possibly associated with longer protection, is recommended in a two dose schedule to be given at two weeks apart. Large scale cholera outbreaks often affect vulnerable populations with limited access to care. Strict dosing schedules can create further logistical barriers, hindering proper vaccine delivery to affected residents returning for their second OCV dose. In this study, 356 participants aged 1 year or older were randomized to receive two doses of OCV at 14 or 28 day intervals, for which vibriocidal immune responses were compared. Similar immune responses were demonstrated between a two and four week OCV dosing schedule, which can increase flexibility when offered as part of a targeted vaccination program. This can further serve to increase adherence and completion of the recommended dosing regimen, as well as providing a platform to increase coverage of other beneficial non-vaccine interventions.

## Introduction

As a disease of poverty and inequity, cholera is often prevalent in areas of compromised sanitation, overcrowded conditions, and poor quality of water supply. An increasing number of longer lasting outbreaks have dramatically impacted the least developed countries (LDCs), including those in Africa, South Asia, and the Hispaniola island region [1]. Living conditions in LDC populations often favor disease transmission and improvements can take a long time to achieve. In these settings, *V*. *cholerae* O1 can cause large, rapidly spreading severe outbreaks that cripple public health systems with already limited medical and financial resources. Many recent epidemics have occurred in highly susceptible and vulnerable populations (Haiti, Zimbabwe, Central and West Africa), where behavioral, social, and environmental factors, as well as lower background exposure to cholera have contributed to increased duration and severity of the outbreaks [2]. Effective interventions combining surveillance, treatment, and improving water, sanitation, and hygiene (WASH) measures are paramount. Vaccination can complement these preventive and control strategies in areas of endemic disease or areas at risk for outbreak [3]. Recently, a killed, bivalent oral cholera vaccine (OCV) has been prequalified and recommended for use by the WHO. Still, this OCV has not been widely implemented in endemic areas and its use is limited to areas with established or imminent outbreaks.

Safety and immunogenicity of this OCV has been demonstrated in Vietnam, India, and Bangladesh [4–6]. Seroconversion with serum vibriocidal antibodies following vaccination was found to be lower in hyper-endemic areas (India) compared to less endemic areas (Vietnam). When participants with only low baseline serum vibriocidal titers were analyzed in the Kolkata trial, seroconversion and geometric fold rise were similar in both populations [7]. A large phase three randomized clinical trial (RCT) of the two-dose, killed bivalent OCV demonstrated a cumulative 65% efficacy in endemic populations over five years [8]. Earlier studies with the cholera toxin whole cell O1 vaccine revealed protection for three years in adults and for 6–12 months in young children [9–11]. In Kolkata, a RCT evaluating immune responses of the bivalent killed whole cell OCV without cholera toxin B (Shanchol, Shantha Biotechnics Limited) in adults and children found robust responses to a first dose but no further rise following the second dose [12]. This observed blunted immune response following the second dose may be due to the increased LPS content, as compared with older versions of killed OCV. Proposed mechanisms of the blunted immune response include blocking of subsequent antibody production by the increased LPS or a booster like effect occurring after the first dose due to recurrent natural exposure in an endemic setting.

Some questions still remain unanswered with regards to the most optimal dosing regimen to assist the effective deployment of OCV in field conditions. An alternate 28 day interval could facilitate inclusion of OCV into a routine immunization schedule in cholera endemic regions. No significant difference in immune response following a four week schedule may further support the hypothesis of whether adequate protection can be offered by a single dose in endemic areas—this is currently being assessed in a large, placebo controlled RCT in Bangladesh. We aimed to assess if immune responses in a prolonged 28 day dosing interval is non-inferior to the standard 14 day schedule.

## Methods

### Study Design

This was a double-blind, RCT conducted at the Clinical Trials Unit of the National Institute of Cholera and Enteric Diseases (NICED), Kolkata, India. Recruitment, dosing and follow up were completed between January-December 2011. The study was performed in the cholera endemic urban slums of Kolkata with similar access to water, sanitation, and health care throughout the study area. Healthy males and non-pregnant females aged ≥1 year were recruited. Exclusion criteria consisted of serious chronic disease, pregnancy, immune-compromised conditions, gastrointestinal disease, antibiotic usage in the past 14 days, or previous receipt of cholera vaccine. Potential participants with acute illness or fever had dosing deferred pending recovery.

The objectives of this trial were to compare safety and serum vibriocidal antibody responses in participants receiving two OCV doses either 14 days or 28 days apart. The primary endpoint was the proportion of participants exhibiting four-fold or greater rises in serum vibriocidal antibody titers, 14 days following the second dose relative to baseline. Secondary endpoints included measurement of geometric mean titers of serum vibriocidal antibody at the above time points. Safety of the vaccine was also evaluated throughout the follow up period.

### Ethics Statement

Written informed consent was obtained by study physicians for all adults and parents/guardians of participating children, as well as written assent for 11–17 year old participants. The trial protocol was approved by the Scientific and Ethics Committee of NICED and the International Vaccine Institute (IVI). Independent safety monitoring was conducted, with external monitoring & GCP audits performed by Shantha Biotechnics Limited. This trial was registered in India (CTRI/2010/091/002807) and clinicaltrials.gov (NCT 01233362). All data from trial volunteers used for analysis was anonymized.

### Study Procedures and Definitions

The study vaccine (Shanchol) consisted of 600 ELISA units of LPS of *V*. *cholerae* O1 El Tor Inaba; 300 ELISA units of LPS each of *V*. *cholerae* O1 classical Ogawa, 300 ELISA units of LPS of *V*. *cholera* O1 Inaba and 600 ELISA units of LPS of *V*. *cholerae* O139. Placebo vials contained *E*. *coli* K12 cells, whose appearance was identical to the study vaccine. Dosing of the study agent was administered as in Table 1. Both placebo and vaccine were packaged as liquid formulations in identical vials containing 1.5 mL doses and were stored at 2–8°C. The study agent was given in two doses separated by a two week or a four week interval and administered by oral syringe, after which each participant was offered a cup of water. Participants were observed in the trials unit for 30 minutes following dosing, as well as for 3 days after each dosing. During each follow up day, study physicians conducted a structured interview regarding the participant’s overall health and any occurrence of adverse events. Diarrhea was defined as three or more loose or liquid stools in a 24 hour period. Blood samples were obtained prior to the first dose and 14 days after each study agent dose. Sera were separated and stored at -70°C until paired testing was performed. The microtiter technique was used to detect serum vibriocidal antibodies to *V*. *cholerae* O1 El Tor Inaba, O1 Ogawa, and O139 [13].

**Table 1:** Dosing schedule for participants.

Interval group	Number of participants	Day 0	Day 14	Day 28
14 day interval	178 participants (89 adults, 89 children)	Vaccine	Vaccine	Placebo
28 day interval	178 participants (89 adults, 89 children)	Vaccine	Placebo	Vaccine

Dose 1 of vaccine was given on day 0 in both groups, whereas dose 2 was given either on day 14 or day 28

### Randomization and Masking

Participants were stratified by age group (1–5y, 6–10y, 11–17y, and ≥18y). Randomization numbers were generated in blocks of at least four, which included equal numbers of each arm, to ensure that balance between treatments was maintained. These lists were prepared by a statistician not involved in the study. Study agents were pre-labeled by Shantha personnel, who were not involved in the conduct or monitoring of the trial. All study staff and participants were blinded to treatment assignment for the duration of the study.

### Statistical Methods

Sample size calculation was driven by seroconversion after two doses under 14 day and 28 day dosing intervals. Among participants, we assumed 45% seroconversion in adults and 80% in children after 2 doses. If the seroconversion rate in the 28 day dosing interval is no less than 20% than that in the 14 day dosing interval, it will be considered to be non-inferior. This threshold was selected based on seroconversion rates and their corresponding lower bounds of the one tailed 95% confidence interval from previous studies using the same vaccine in the same setting[5, 12]. Assuming a one tailed α = 0.05, 80% power, a 15% drop-out rate, and using the score method of non-inferiority test [14], a total of 89 participants per study group had been considered. Thus, a total of 356 participants were targeted, 178 in each dosing regimen.

Data were entered in a web-based data capture system and analyses were performed in SAS 9·3 (SAS Institute, Cary NC). Analyses for comparisons of dichotomous outcomes such as adverse events and seroconversion were performed with the chi-square test or Fisher’s exact test if cell counts were sparse. For comparisons of vibriocidal titers, Student’s t-test was performed using the pooled or Satterthwaite method depending on whether the variances were equal or not. Nonparametric Wilcoxon rank-sum test and Kolmogorov-Simirnov test were performed when data were not normally distributed. Comparisons of the primary outcomes, vibriocidal seroconversion were evaluated with one-tailed 95% confidence intervals using the Wilson Score method[15]. Statistical evaluations of all other comparisons were two tailed.

## Results

### Participant recruitment and baseline data

**Fig 1:**
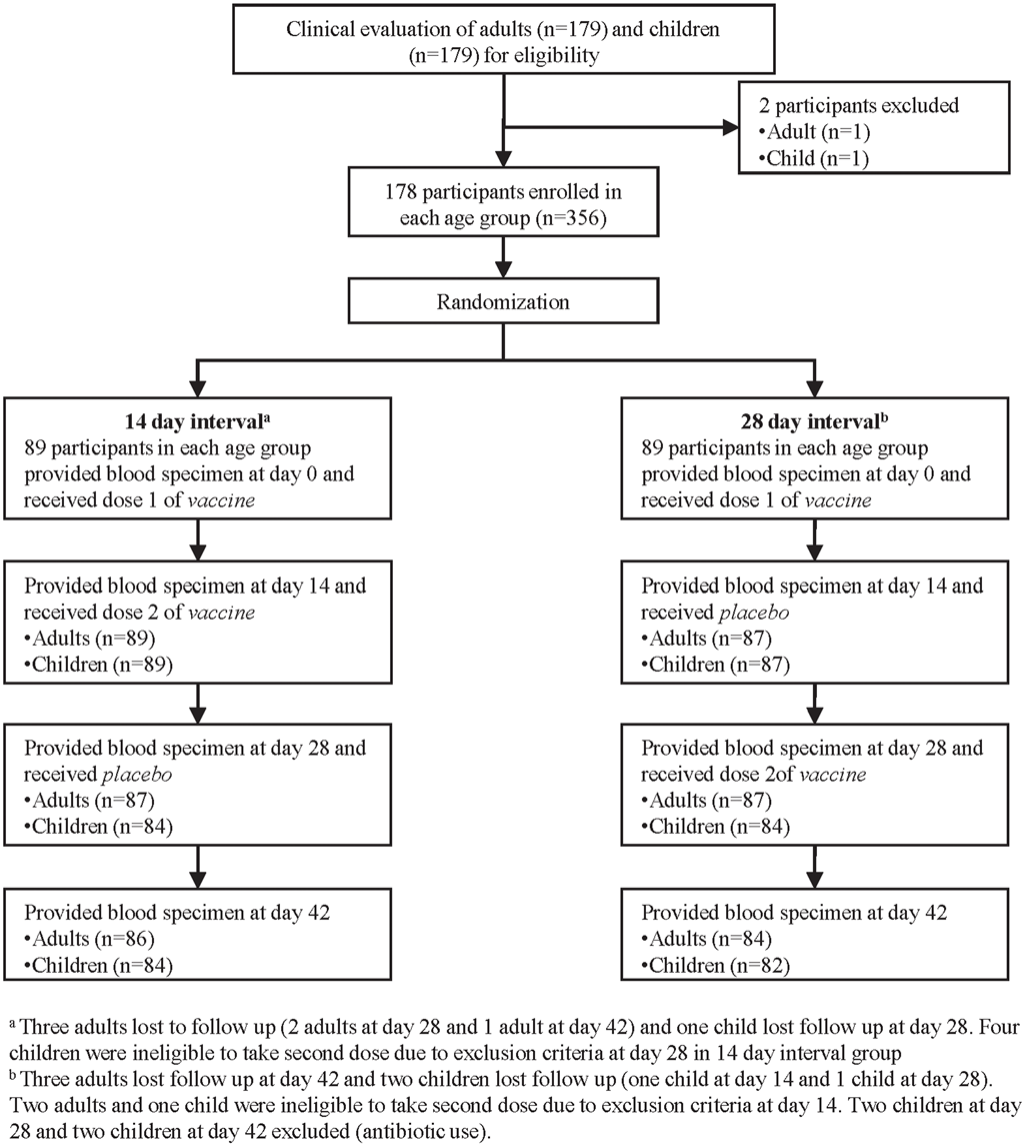
Flowchart of adult and children participants in the study.

Recruitment of participant flow is illustrated in Fig. 1A total of 356 participants (178 children, 178 adults) were recruited from January 2010 to October 2011. Among eligible participants, 86/89 adults (96.6%) and 84/89 children (94.4%) in the 14 day interval arm and 84/89 adults (94.4%) and 82/89 children (92.1%) of the 28 day interval arm took all three doses of the assigned study agent and provided all four blood samples. A total of 20 participants (5.6%) were lost to follow up or were found to be ineligible to continue following study visit screening. There were no significant differences in demographic characteristics between intervention arms among each age group (Table 2).

**Table 2:** Demographic characteristics of study participants.

	Adults			Children		
Characteristics	14 day interval	28 day interval	p value	14 day interval	28 day interval	p value
	n = 89	n = 89		n = 89	n = 89	
Age (years)						
Mean (SD)	28.4 (6.1)	27.4 (5.9)	0.260	8.96 (4.11)	8.76 (3.85)	0.740
Median	28.3	26.7	0.250	8.50	8.31	0.855
Sex						
Male (%)	26 (29)	36 (40)	0.116	39 (44)	45 (51)	0.368
Female (%)	63 (71)	53 (60)		50 (56)	44 (49)	

### Outcomes

All participants randomized in the study were included in safety outcome analysis. No statistically significant differences in the rates of adverse events between each intervention group were noted (Table 3). A total of 10 adverse events (AE) were reported within 3 days of either dose. The most commonly reported AEs were fever (n = 3), general ill feeling (n = 2), vomiting, diarrhea, and headache (with n = 1 each), with no statistically significant differences between children or adults. No serious adverse events were reported during the trial.

**Table 3:** Solicited adverse events among adults and children following 14 and 28 day dosing intervals.

	14 day interval	28 day interval		p value
Number of AEs within 3 days after first vaccine dose	2^a^		6^b^	0.39
Number of AEs within 3 days after second vaccine dose	2^c^		0	0.32
Number (%) of participants with ≥ 1 AEs 3 days following dosing regimen	2/178 (1.1%)		3/178 (1.7%)	1
Number (%) of participants with SAEs 28 days following dosing regimen	0/178 (0%)		0/178 (0%)	--

^a^ mild fever (n = 1) and mild headache (n = 1)

^b^ mild diarrhea (n = 1), mild nausea (n = 1), mild general ill feeling (n = 1), mild fever (n = 2), mild vomiting (n = 1)

^c^ moderate diarrhea (n = 1), mild general ill feeling (n = 1)

A per protocol analysis was conducted for immunogenicity data, including 336 participants who completed all planned study visits. Immune responses to *V*. *cholerae* O1 Inaba, O1 Ogawa, and O139 following administration of two doses of vaccine in a 28-day schedule were non-inferior to those of a 14 day schedule, as the difference measured was greater than the pre-defined cut-off of-20% (Tables 4,5). No significant difference between dosing schedules was observed in percentage of seroconversion after the first or second dose. Baseline vibriocidal geometric mean titers (GMT) to O1 Inaba ranged from 94 to 275 in adults and from 29 to 140 in children. The geometric mean fold (GMF) rise was higher in children (ranging from 7.5–26.9) than in adults (3.4–6.4). No statistically significant difference was noted between intervention arms in seroconversion or geometric fold rise. The GMF rise from baseline was higher for O1 Inaba in adults, after receipt of the first dose (6.8 and 8.9 respectively in the 14 and 28 day interval arms) compared to receipt of the second dose (4.6 and 4.7 respectively). In children the responses were more pronounced with GMF rise from baseline after first dose in both the arms being 29.7 and 20.8 respectively. The GMF rise after second dose was 17.5 and 10.7 respectively. Rise in titers to *V*. *cholerae* O1 were higher among individuals with lower baseline vibriocidal titers, as seen in Table 5.This magnified response was likely due to the lower baseline GMT observed in children, suggesting lower natural exposure. Adults with baseline GMTs lower than the median (<160) demonstrated high GMF rise (>10) and seroconversion (~85%) in both interval groups, which were markedly higher than adults with higher baseline GMTs (Table 6). Comparable results were noted in children, although median baseline titers were lower (80). There was a significantly higher GMF rise in children aged 1–5 years old in the 14 vs 28 day interval groups (34.7 vs 10, p = 0.01), though no significant difference in seroconversion was noted. This difference is most likely explained by the significantly higher baseline GMT detected in 1–5 year olds between the 14 and 28 day interval group (14.1 vs 69.6, p = 0.01, S1 Table). No other significant differences were noted in any other age group. When controlling for baseline GMT, a multiple linear regression model of log transformed titers did not find any significant difference between the two dosing intervals (-0.13 dosing interval effect comparing the 28 day interval to the 14 day interval, p = 0.33, S2 Table). Similar observations were also found for O1 Ogawa. Following the second dose, adults demonstrated GMFr of 4.1 with 45% seroconversion and 3.8 with 49% seroconversion to O1 Ogawa in 14 and 28 day interval groups (Tables 4, 5). Children exhibited GMFr of 11.1 with 73% seroconversion and 7.8 with 72% seroconversion. As with previous trials in Vietnam, India, and Bangladesh, immunogenicity against O139 was poor in both schedules [4–6].

**Table 4:** Vibriocidal antibody titers and proportion of ≥ 4 fold rise from baseline GMT in adults.

Adults		O1 Inaba			O1 Ogawa			O139		
		14 day interval (n = 86)	28 day interval (n = 84)	p value	14 day interval (n = 86)	28 day interval (n = 84)	p value	14 day interval (n = 86)	28 day interval (n = 84)	p value
**Baseline**	GMT^a^ (95% CI)	191.0 (133, 275)	143.7 (94.2, 219)	0.31	364 (252, 526)	359 (244, 528)	0.96	5.4 (4.2, 6.9)	4.9 (3.9, 6.3)	0.62
**14 days after first vaccine dose**	GMT^a^ (95% CI)	1301 (1032, 1639)	1280 (954, 1717)	0.93	2076 (1660, 2596)	2083 (1636, 2651)	0.98	9.9 (7.5,13.2)	9.6 (7.2,12.9)	0.85
	GMF rise^b^ (95% CI)	6.8 (5.0, 9.3)	8.9 (6.0, 13.3)	0.30	5.7 (4.1, 8.0)	5.8 (4.2, 7.9)	0.94	1.8 (1.5, 2.3)	1.9 (1.5, 2.5)	0.77
	N (%) who seroconverted^c^	59 (69%)	55 (66%)	0.66	48 (56%)	52 (62%)	0.42	22 (26%)	23 (27%)	0.79
	95% CI ^d^	58.2%– 77.4%	54.8%- 74.8%		45%-66%	51%-72%		18%-36%	19%-38%	
**14 days after second vaccine dose**	GMT^a^ (95% CI)	876 (687, 1117)	678 (529, 868)	0.14	1492 (1219, 1825)	1356 (1089, 1689)	0.53	8.4 (6.4,11.1)	8.9 (6.5,12.1)	0.80
	GMF rise^b^ (95% CI)	4.6 (3.4, 6.2)	4.7 (3.4, 6.4)	0.90	4.1 (3.0, 5.6)	3.8 (2.9, 4.9)	0.70	1.6 (1.3, 1.8)	1.8 (1.4, 2.3)	0.33
	No (%) who seroconverted^c^	47 (55%)	49 (58%)	0.63	39 (45%)	41 (49%)	0.65	17 (20%)	17 (20%)	0.94
	95% CI^d^	44.2%- 64.7%	47.7%- 68.3%		35%-56%	38%-59%		13%-29%	13%-30%	
N (%) who seroconverted following either first or second dose		59 (69%)	56 (67%)	0.79	50 (58%)	53 (63%)	0.51	23 (27%)	25 (30%)	0.66
Proportion difference (Lower boundary of one-tailed 95% CI)^e^		--	3% (-8.6%)	--	--	4% (-8.9%)	--	--	0.5% (-9.9%)	--

^b^Geometric mean-fold rise from baseline to 14 days after first dose or from baseline to 14 days after second dose

^c^ # with ≥4 fold rise in titers from baseline to 14 days after first dose or from baseline to 14 days after second dose

^d^ 95% confidence intervals using Wilson Score method

^e^ Primary endpoint. Difference in seroconversion rates after second dose were calculated by subtracting 14 day interval from 28 day interval. The 28 days interval group is non-inferior to the 14 day interval group as the lower limit of the proportion difference is greater than pre-defined cut-off (-20%)

**Table 5:** Vibriocidal antibody titers and proportion of ≥ 4 fold rise from baseline GMT in children.

Children		O1 Inaba			O1 Ogawa			O139		
		14 day interval (n = 84)	28 day interval (n = 82)	p value	14 day interval (n = 83)	28 day interval (n = 81)	p value	14 day interval (n = 82)	28 day interval (n = 80)	p value
**Baseline 14 days after first vaccine dose**	GMT^a^ (95% CI)	47.2 (29.1, 76.5)	88.5 (56.1, 140)	0.06	124.5 (75.7, 205)	131 (77.5, 223)	0.88	3.7 (3.0, 4.5)	3.7 (2.9, 4.6)	0.95
	GMT^a^ (95% CI)	1402 (894.1, 2197)	1841 (1267, 2676)	0.36	2335 (1656, 3294)	2049 (1423, 2952)	0.60	10.9 (7.8,15.4)	12.3 (9,16.9)	0.62
	GMF rise^b^ (95% CI)	29.7 (18.2, 48.6)	20.8 (13.5, 32)	0.28	18.7 (11.9, 29.5)	15.6 (10.3, 23.5)	0.55	3 (2.2, 4.1)	3.3 (2.5, 4.4)	0.62
	N (%) who seroconverted^c^	72 (86%)	73 (89%)	0.52	63 (75%)	65 (79%)	0.51	34 (40%)	36 (44%)	0.65
	95% CI ^d^	76.7%- 91.6%	80.4%- 94.1%		65%-83%	69%-87%		31%-51%	34%-55%	
**14 days after second vaccine dose**	GMT^a^ (95% CI)	827 (553, 1235)	952 (676, 1341)	0.60	1380 (992, 1920)	1025 (702, 1496)	0.24	7.8 (5.7,10.7)	7.2 (5.3, 9.8)	0.70
	GMF rise^b^ (95% CI)	17.5 (11.4, 26.9)	10.7 (7.5, 15.5)	0.09	11.1 (7.5, 16.4)	7.80 (5.2, 11.6)	0.21	2.1 (1.7, 2.7)	1.9 (1.5, 2.43)	0.57
	N (%) who seroconverted^c^	67 (80%)	63 (77%)	0.65	61 (73%)	59 (72%)	0.92	23 (27%)	21 (26%)	0.80
	95% CI^d^	70%- 87%	66.6%- 84.6%		62%-81%	61%-81%		19%-38%	17%-36%	
N (%) who seroconverted following either first or second dose		74 (88%)	75 (91%)	0.47	67 (81%)	69 (85%)	0.45	37 (45%)	40 (50%)	0.53
Proportion difference (Lower boundary of one-tailed 95% CI)^e^		--	-3% (-13.6%)	--	--	-1% (-12.1%)	--	--	-1.8% (-13%)	--

^a^Geometric mean reciprocal titers

^b^Geometric mean-fold rise from baseline to 14 days after first dose or from baseline to 14 days after second dose

^c^ # with ≥4 fold rise in titers from baseline to 14 days after first dose or from baseline to 14 days after second dose

^d^ 95% confidence intervals using Wilson Score method

^e^ Primary endpoint. Difference in seroconversion rates after second dose were calculated by subtracting 14 day interval from 28 day interval. The 28 days interval group is non-inferior to the 14 day interval group as the lower limit of the proportion difference is greater than pre-defined cut-off (-20%)

**Table 6:** Geometric mean fold rises to *V*. *cholerae* O1 Inaba and number who develop ≥ 4 fold rises from baseline after 14 and 28 day dosing intervals.*

	GMF—rise from baseline			No. with ≥ 4 fold rise from baseline		
	14 day interval	28 day interval	p value	14 day interval	28 day interval	P value
**All age groups**						
All (n = 336)	8.9 (n = 170)	7.1 (n = 166)	0.23	114/170 (67.1)	112/166 (67.5)	0.94
Baseline vibriocidal ≤ 160 (n = 202)	20.9 (n = 103)	16.3 (n = 99)	0.29	91/103 (88.3)	88/99 (88.9)	0.90
Baseline vibriocidal > 160 (n = 134)	2.4 (n = 67)	2.1 (n = 67)	0.27	23/67 (34.3)	24/67 (35.8)	0.86
**1–5 years**						
All (n = 51)	34.7 (n = 26)	10.0 (n = 25)	0.01	24/26 (92.3)	21/25 (84.0)	0.42
Baseline vibriocidal ≤ 80 (n = 32)	50.2 (n = 20)	25.4 (n = 12)	0.25	19/20 (95.0)	11/12 (91.7)	1.00
Baseline vibriocidal > 80 (n = 19)	10.1 (n = 6)	4.2 (n = 13)	0.10	5/6 (83.3)	10/13 (76.9)	1.00
**6–10 years**						
All (n = 56)	13.8 (n = 28)	10.8 (n = 28)	0.64	19/28 (67.9)	22/28 (78.6)	0.37
Baseline vibriocidal ≤ 80 (n = 29)	67.5 (n = 13)	26.9 (n = 16)	0.22	11/13 (84.6)	15/16 (93.8)	0.57
Baseline vibriocidal > 80 (n = 27)	3.5 (n = 15)	3.2 (n = 12)	0.80	8/15 (53.3)	7/12 (58.3)	0.80
**11–17 years**						
All (n = 59)	12.1 (n = 30)	11.4 (n = 29)	0.90	24/30 (80.0)	20/29 (69.0)	0.33
Baseline vibriocidal ≤ 80 (n = 32)	28.7 (n = 19)	46.5 (n = 13)	0.45	18/19 (94.7)	12/13 (92.3)	1.00
Baseline vibriocidal > 80 (n = 27)	2.7 (n = 11)	3.7 (n = 16)	0.37	6/11 (54.5)	8/16 (50.0)	0.82
**18+ years**						
All (n = 170)	4.6 (n = 86)	4.7 (n = 84)	0.90	47 /86 (54.7)	49 /84 (58.3)	0.63
Baseline vibriocidal ≤ 160 (n = 90)	10.4 (n = 43)	10.9 (n = 47)	0.86	36 /43 (83.7)	40 /47 (85.1)	0.86
Baseline vibriocidal > 160 (n = 80)	2.0 (n = 43)	1.6 (n = 37)	0.15	11 /43 (25.6)	9 /37 (24.3)	0.90

* Median baseline titers were used for each age group (160 cut off for adults, 80 for children)

^a^ Geometric mean fold (GMF) rise from baseline to 14 days after dose 2

^b^ Number of participants with ≥ 4 fold rise in titers from baseline to 14 days after dose 2

^c^ p values comparing GMF-rise from baseline to 14 days after dose 2 between 14 days and 28 days interval groups

^d^ p values comparing ≥4-fold rise from baseline to 14 days after dose 2 between 14 days and 28 days interval groups

## Discussion

The results of our study support flexibility in dosing Shanchol in endemic settings, where strict schedules may be difficult to adhere to. As with any immunogenicity findings, vibriocidal antibodies do not truly reflect a protective response and, at best, are an indirect correlate of protection that is not absolute. While only a field trial can determine true effectiveness of altered dosing regimens, interpreting this data in light of the existing immunogenicity and clinical efficacy data in the same setting may provide a foundation for policy makers to ease implementation of OCV as part of a control strategy for cholera.

Both schedules were well tolerated by all recipients with comparable safety profiles between either group. Our findings were compatible with previous studies that revealed that high baseline vibriocidal titers were associated with reduced post-vaccination serum vibriocidal antibody responses[5, 16]. Higher baseline titers found among participants were most likely due to prior exposure to *V*. *cholerae* since the area is cholera-endemic and the population had not earlier received cholera vaccine. The first dose of the vaccine may have elicited memory immune responses among previously exposed individuals resulting in a rise in vibriocidal titers with no further rises after the second dose. In children, the baseline vibriocidal titers were lower, suggesting lower earlier exposure in this age group. Lower baseline titers were associated with higher GMF rise increases following vaccination, with a higher percentage of responders in this age group, though the clinical significance of this finding is unclear. Although it is possible that these results reflect chance, a recurrent theme of immune differences in children under five years of age relating to OCV does occur, and it is possible that in this sub-population that there may be a difference between the two regimens.

Our study confirms earlier findings that the two-dose regimen of the killed whole-cell OCV is safe, well-tolerated, and immunogenic [17]. Vibriocidal responses to O1 Inaba were higher in both adults and children following the first dose, as compared to the second dose, with GMFr rises higher in children, likely related to the inverse relation of baseline serum titers mentioned above. Whether the lower responses to O139 indicate that the vaccine elicits poorer responses to O139 or if this reflects differences in assay sensitivity remains an aspect that needs to be explored with additional scientific data. *V*. *cholerae* O139 continues to be infrequently isolated from environmental samples but has not been responsible for any large outbreak in the past 10 years. The lack of circulating O139 strains could be a possible factor for the lower immune response to O139 antigen in the vaccine.

Serum vibriocidal antibody responses were shown to be no higher following a second dose, when compared to levels after the first dose. This contrasts with the older generation killed OCV (Dukoral), for which serum titers increased further after the second dose[18]. The current reformulated killed whole cell vaccine (Shanchol) elicits higher serum vibriocidal responses than the older version of Dukoral. It exhibits no augmentation of these responses after the second dose as compared with the first, perhaps because it has an approximately two times higher LPS content than the older vaccine[4]. This marked difference in magnitude and pattern of immune responses motivated the current evaluation of whether extending the interval between doses has an impact on the vibriocidal response. While extending the dosing interval did not raise immune responses to the second dose, the mechanism behind this observed lack of boosting remains unclear. Since the vibriocidal antibody response does not truly reflect protection, and at best is an indirect correlate of protection, our immunogenicity results are not sufficient to support a hypothesis that a single dose regimen may confer similar efficacy as two doses. Nevertheless, the similarity of immune responses to shorter versus longer inter-dose intervals provides some reassurance that flexibility in dosing, particularly extending the intervals beyond 14 days, will not vitiate vaccine response.

Comparable immune responses between different dosing regimen schedules would support additional uses of vaccination as part of a comprehensive strategy. In endemic settings, policy makers could entertain extending the dose interval to 1 month, which could ease delivery by facilitating national routine immunization strategies and linking OCV with other health interventions to populations in high risk regions. These results may be of particular interest in complex outbreaks, such as those seen following a natural disaster. A reactive vaccination strategy provides vaccine following a cholera outbreak to prevent further disease transmission with hopes of shortening outbreak duration. It relies on getting the first dose to affected populations as soon as possible. After the first dose is distributed, one month interval could allow the focus to return to stabilization of infrastructure and water sanitation. This is pertinent to a post disaster context in resource limited areas, which can be a common scenario for cholera outbreaks in both endemic and non-endemic areas (Indonesia tsunami, Haitian earthquake, Pakistan floods). Since this study was conducted in an endemic area and a population with pre-existing vibriocidal antibodies, the results may be different than what can be expected from non-endemic areas. Evaluations of a longer dosing interval in these settings are needed since immunogenicity and overall vaccine impact may likely be impacted by recurrent exposure, or ‘natural boosting’. With no further rise in seroconversion rates after a second dose, efforts to evaluate a efficacy of a single dose regimen in a clinical field trial is underway to evaluate its potential use in an epidemic setting [19]. From a programmatic standpoint, additional exploration into serum and gut responses when spacing out the dosing interval even further may broaden our knowledge on public health benefits with regards to the amount and duration of clinical protection offered by this OCV.

Cholera remains a major global health concern and is an important threat to most developing countries, especially in areas where overcrowding and poor sanitation are common. Large outbreaks often involve populations affected by natural disasters or those displaced by war, where there is inadequate sewage disposal and contaminated water. In spite of current WHO support for use of OCV as part of a prevention and control package for cholera endemic areas, the international community is still exploring the best methods to implement these recommendations. Flexibility with the administration of two doses over one month could ease logistical requirements in a complex outbreak setting, allowing for stabilization of community infrastructure, as well as linking vaccination with other vital community interventions, resulting in the enhanced delivery of OCV. By demonstrating similar immunologic responses to different dosing regimens, with no additional safety risk, further operational research testing even longer inter-dose intervals could provide helpful answers to improve decision making to fill critical knowledge gaps for vaccination in endemic, epidemic, and outbreak scenarios.

## Supporting Information

S1 Checklist Consort checklist. (PDF)

S1 Protocol A randomized controlled trial to evaluate the immunogenicity of two doses of the modified killed whole cell oral cholera vaccine (WC-ocv) under two alternative vaccination schedules (Version 5.0, April 2012). (DOCX)

S1 Table Vibriocidal antibody titers and proportion of ≥ 4 fold rise from baseline GMT to *V*. *cholerae* O1 Inaba (1–5 years, 6–17 years). (DOCX)

S2 Table Multiple regression analysis of dosing interval effect controlling for baseline GMT. (DOCX)
