# Supplementary material for: Flexibility of Oral Cholera Vaccine Dosing—A Randomized Controlled Trial Measuring Immune Responses Following Alternative Vaccination Schedules in a Cholera Hyper-Endemic Zone
Source: PLoS Negl Trop Dis. 2015 Mar 12;9(3):e0003574. doi: 10.1371/journal.pntd.0003574 (PMC4357440; doi:10.1371/journal.pntd.0003574)
Supplement: S1 Protocol — (DOCX) [file pntd.0003574.s002.docx]

**a randomized controlled trial to evaluate the immunogenicity of two doses of the modified killed whole cell oral cholera vaccine (WC-ocv) under two alternative vaccination schedules**

| **COLLABORATORS:**  **Sponsor:** | Indian Council of Medical Research, Ansari Nagar, New Delhi 110029, India, Telephone (91-11) 2651-7204, Fax (91-11) 2686-8662  National Institute of Cholera and Enteric Diseases, Beliaghata, Kolkata 700010, West Bengal, India, Telephone (91-33) 2353-7519, Fax (91-33) 2350-5066  Shantha Biotechnics PVT.LTD., Serene Chambers, 3rd Floor, Road No. 7, Banjara Hills, Hyderabad 500 034 India, Telephone (91-40) 23543010, Fax (91-40) 23541713  International Vaccine Institute, San 4-8 Nakseongdae – dong, Kwanak, Seoul, 151-818 Korea Telephone (82-2) 872-2801, Fax (82-2) 872-2803 |
| --- | --- |
| **Investigational product:** | Killed bivalent (O1 and O139) whole cell oral cholera vaccine |
| **Control Product:** | Heat-killed *Escherichia coli* K12 placebo |
| **Principal Investigator**: | Dr. Dipika Sur, Deputy Director, National Institute of Cholera and Enteric Diseases, Kolkata 700010, West Bengal, India, Telephone (91-98) 3101-9515 |
| **Co-Investigators :** | Dr. Suman Kanungo, Dr. Byomkesh Manna, Dr.Mihir Kumar Bhattacharya, Dr Ranjan Nandy |
| **IVI Investigators** | Sachin N. Desai, Binod K. Sah, Rodney Carbis, Thomas F. Wierzba |
| **Safety MONITOR:** | Dr.Dilip Mahalanabis, Director, Society for Applied Studies, Kolkata, West Bengal, India, Telephone (91-98) 3051-5374 |
| **DATA MANAGEMENT:** | Dr. Mohammad Ali, Mr. Mahesh K. Puri |
| **statisticianS:** | Ms. Deok Ryun Kim |
| **Date:** | April 2012 |
| **PROTOCOL version:** | CR-WC-05 version 5.0 |

-ii-

**Table of Contents**

1. INTRODUCTION
   1. Background 1
   2. Study Rationale 2
2. STUDY OBJECTIVES
   1. Main Study
      1. Primary Objectives 2
      2. Secondary Objectives 3
   2. Additional Exploratory Study Objective 3
3. STUDY DESIGN
   1. Endpoints 3
      1. Primary Endpoints
      2. Secondary Endpoints
      3. Additional Exploratory Study Endpoints 3
   2. Type of Study 4
   3. Random Allocation 4
   4. Blinding and Code Breaking Procedures 4
4. RERCRUITMENT AND ENROLMENT
   1. Recruitment 5
   2. Inclusion Criteria 5
   3. Exclusion Criteria 5
   4. Subject Withdrawal During the Study 6
   5. Identification of Subjects 6
   6. Duration of the Study Period for One Subject 7
5. STUDY AGENTS
   1. Vaccine 7
   2. Placebo 8
   3. Packaging and Coding 8
   4. Study Agents Accountability 8
   5. Storage Conditions 9
   6. Administration 9
6. STUDY PROCEDURES
   1. Scheduling and Description of Observations and Visits 9
   2. Assessment after Vaccination
      1. Follow-up for Adverse Events 11
      2. Follow-up for Serious Adverse Events 12
      3. Blood Draws 12
      4. Stool Samples 13
      5. Sample Storage 13
   3. Laboratory Procedures 13
7. ADVERSE EVENTS
   1. Definitions 14
   2. Assessment of Causality 15
   3. Assessment of Severity 15
   4. Reporting of Serious Adverse Events 16
8. DATA COLLECTION AND MANAGEMENT PROCESDURES
   1. Case Report Forms (CRF) 17
   2. Source Documentation 17

-III-

- 1. Data Management 17

1. STATISTICAL CONSIDERATIONS
   1. Sample Size Calculation 18
   2. Analysis Plans 18
2. MONITORING, AUDITING, INSPECTION
   1. Responsibilities of the Investigator (s) 19
   2. Responsibilities of the IVI Coordinator (s) 20
   3. Responsibilities of the Safety Monitor 20
3. ETHICAL AND REGULATORY STANDARDS
   1. Ethical Principles/Laws and Regulations 20
   2. Potential Risks and Risk Minimization 21
   3. Informed Consent and Assent 21
   4. Institutional Review Committee (IRB)/Ethics Committee (EC) 21
4. ADMINISTRATIVE ASPECTS
   1. Record Retention 22
   2. Publications 22
   3. Study Funding 23

13. REFERENCES 24

-IV-

**List of Abbreviations**

AE Adverse event

ALS Antibody in Lymphocyte Supernatant

CI Confidence Interval

CRF Case Report Form

EC Ethics Committee

EU Enzyme Linked Immunosorbent Assay (ELISA) Units

ICH – GCP International Conference on Harmonization- Good Clinical Practices

GMP Good Manufacturing Practices

GMT Geometric Mean Titers

IgA Immunoglobulin A

IRB Institutional Review Board

IVI International Vaccine Institute

LPS Lipopolysaccharide

NICED National Institute of Cholera and Enteric Diseases

OCV Oral Cholera Vaccine

OMP Outer Membrane Protein

SAE Serious Adverse Event

WHO World Health Organization

**-V-**

**STUDY SUMMARY**

| **TITLE** | | A randomized controlled trial to evaluate the immunogenicity of two doses of modified killed whole cell oral cholera vaccine (WC-OCV) under two alternative vaccination schedules |
| --- | --- | --- |
| **STUDY OBJECTIVES** | | **Main study:**  **Primary objective:**  To compare vibriocidal immune responses to the modified killed WC-OCV when given as two doses, 14 and 28 days apart, in healthy, non-pregnant adult and children volunteers  **2) Secondary objectives:**  To confirm the safety of two doses of modified killed WC-OCV in healthy, non-pregnant adult and children volunteers  **Additional Exploratory Study:**  To explore feasibility of using other immunological indicators in determining the immunogenicity of modified killed WC-OCV in an additional cohort of 30 subjects:  (Fecal antibody to LPS, OMP and LPS-specific microELISPOT, IgA to LPS endpoint ELISA and ALS assays) |
| **STUDY DESIGN** | | Individually randomized, double-blind, controlled trial in healthy, non-pregnant adults and children allocated to the following intervals:   \| Interval \| Size \| Day 0 \| Day 14 \| Day 28 \| \| --- \| --- \| --- \| --- \| --- \| \|  \|  \|  \|  \|  \| \| 14 day interval \| 89 adults \| Vaccine \| Vaccine \| Placebo \| \| 89 children \| Vaccine \| Vaccine \| Placebo \| \| 28 day interval \| 89 adults \| Vaccine \| Placebo \| Vaccine \| \| 89 children \| Vaccine \| Placebo \| Vaccine \|  \| **Additional cohort for immunological assessment**: Individually randomized, double-blind, controlled trial in healthy, non-pregnant adults allocated to the following intervals: Interval \| Size \| Day 0 \| Day 14 \| Day 28 \| \| --- \| --- \| --- \| --- \| --- \| \|  \|  \|  \|  \|  \| \| 14 day interval \| 15 adults \| Vaccine \| Vaccine \| Placebo \| \| 28 day interval \| 15 adults \| Vaccine \| Placebo \| Vaccine \| |
| **STUDY SUBJECTS** | | Healthy, non pregnant adults aged **18 years and above**  Healthy children aged 1 – 17 years. |
| **SAMPLE SIZE** | | Total: 386 subjects (208 adults and 178 children)   - Main study group: 356 subjects (178 adults and 178 children) - Additional cohort for immunologic assays: 30 adults subjects |
| **STUDY AGENTS** | | - Modified Killed bivalent (O1 and O139) whole cell oral cholera vaccine (WC-OCV) - Heat-killed *Escherichia* coli K12 placebo |
| **STUDY END-POINTS** | **Primary:**  Proportion of subjects exhibiting 4-fold or greater rises in titers of serum vibriocidal antibodies, relative to baseline, 14 days after last dose of study agent in each dose-interval group  **Secondary:**   1. Proportion of subjects exhibiting 4-fold or greater rises in titers of serum vibriocidal antibodies, relative to baseline, 14 days after first dose of study agent 2. Geometric mean serum vibriocidal titers at baseline, 14 days after each dose of the study agent, in each dose-interval group. 3. Proportion of subjects given 2 doses of study agent given 14 and 28 days apart with any of the following adverse events:    1. Immediate reactions within 30 minutes after each dose    2. Serious Adverse Events occurring throughout the trial    3. Reactogenicity during three consecutive days following each dose: Headache, vomiting, nausea, abdominal pain/cramps, gas, diarrhea, fever, loss of appetite, general ill feeling       1. Diarrhea is defined as having 3 or more loose/watery stools within a 24 hour period.       2. Fever is defined as having an oral or tympanic temperature of ≥38^o^ C   **Additional Exploratory Study:**  Proportion of subjects given 2 doses of vaccine given 14 and 28 days apart with significant responses to:   1. Fecal antibody to LPS 2. LPS-specific micro ELISPOT 3. IgA to LPS (by ELISA) |  |
| **STUDY PERIOD** | 21 months |  |

**-VII-**

**FLOWCHART (Schedule of visits)**

356 subjects in the main study population (Group A, healthy, non-pregnant adults of aged 18 years and above / Group B, children 1 to 17 years of age):

| **Study Procedure** | Day  0 | Day  1 to 3 | Day 14 | Day  15 to 17 | Day 28 | Day  29-31 | Day  42 |
| --- | --- | --- | --- | --- | --- | --- | --- |
| Informed Consent | X |  |  |  |  |  |  |
| History and Physical Exam | X |  |  |  |  |  |  |
| Screening | X |  | X |  | X |  |  |
| Randomization | X |  |  |  |  |  |  |
| Clinical Evaluation |  |  | X |  | X |  | X |
| Blood Draw - Vibriocidal Assay | X |  | X |  | X |  | X |
| Administration of Study Agents- Cholera Vaccine or Placebo | X |  | X |  | X |  |  |
| Solicited Symptoms & Adverse Event Monitoring | X | X | X | X | X | X |  |
| Adverse Event Monitoring |  |  |  |  |  |  | X |

30 subjects in the additional cohort for assessment of other immunologic tests (Group C, healthy, non-pregnant adults of aged 18 years and above):

| **Study Procedure** | Day  0 | Day  1 to 3 | Day 7 | Day 14 | Day  15 to 17 | Day 21 | Day 28 | Day  29-31 | Day  35 | Day 42 |
| --- | --- | --- | --- | --- | --- | --- | --- | --- | --- | --- |
| Informed Consent | X |  |  |  |  |  |  |  |  |  |
| History and Physical Exam | X |  |  |  |  |  |  |  |  |  |
| Screening | X |  |  | X |  |  | X |  |  |  |
| Randomization | X |  |  |  |  |  |  |  |  |  |
| Clinical Evaluation |  |  |  | X |  |  | X |  |  | X |
| Blood Draw - immunologic assays | X |  | X |  |  | X |  |  | X |  |
| Stool sample collection for fecal antibody assay | X |  | X | X |  | X | X |  | X | X |
| Administration of Study Agents- Cholera Vaccine or Placebo | X |  |  | X |  |  | X |  |  |  |
| Solicited Symptoms & Adverse Event Monitoring | X | X |  | X | X |  | X | X |  |  |
| Adverse Event Monitoring |  |  | X |  |  | X |  |  | X | X |

# INTRODUCTION

## Background

Cholera re-emerged as a global threat to public health in 2006 with a 79% increase in cases compared to the previous year. With increasing numbers of displaced populations living in unsanitary conditions and improvements to sanitation and hygiene unrealistic in the near future, the problem is likely to continue to escalate. Approximately 99% of the reported cholera cases and most of the cholera deaths were from Africa. The case fatality rate (CFR) in Africa was as high as 30% in some high risk areas, and the worldwide CFR increased to 2.7% from 1.7% in 2005 [1]. These figures are believed to be underestimates, with as many as 1 million cases and 100 000 to 130 000 deaths believed to occur each year [2]. Underreporting is assumed to exist due to inconsistencies in the definition of cholera, limitations of surveillance, and fear of international travel and trade sanctions. However, the revised International Health Regulations have been in effect since June 2007, to ensure that trade and economic sanctions will no longer be imposed on countries with cholera. Instead, open reporting of cholera outbreaks is encouraged so that they can be contained in a timely manner [3].

In 2006, India reported 1939 cases of cholera to the World Health Organization (WHO) [1] while states such as West Bengal, Maharashtra, Andhra Pradesh, Tamil Nadu, Karnataka and Delhi have long been reporting outbreaks [4-7]. Since the dramatic appearance of the new serotype O139 Bengal in 1992 in Chennai [8], both *V cholerae* O1 and O139 are present in India.

Provision of safe water and food, establishment of adequate sanitation, and implementation of personal and community hygiene constitute the main public health interventions against cholera. These measures cannot be implemented fully in the near future in most cholera-endemic areas. Improvements to water and sanitation require substantial long-term investments, commitment from the local government and often take years to implement. In the meantime, a safe, effective, and affordable vaccine would be a useful tool for cholera prevention and control [9].

Considerable progress has been made during the last decade in the development of new generation oral vaccines against cholera. These have already been licensed in some countries and in the recent meeting of the WHO Strategic Advisory Group of Experts on immunization in October 2009 recommended the use of oral cholera vaccines in endemic areas and in outbreak settings [9].

A monovalent (anti-O1) killed whole cell oral cholera vaccine (WC-OCV) was developed by Prof Jan Holmgren in Sweden and is now internationally licensed and WHO prequalified. The vaccine consists of inactivated whole cells of *V. cholerae* supplemented with a purified recombinant–DNA derived B-subunit of the cholera toxin. Large scale field trials of the vaccine in Bangladesh and Peru showed that both the killed whole cell vaccine containing the B-subunit, as well as the killed whole cell preparation alone, conferred significant protection for recipients for up to 3-5 years depending on age. An initial protection of 85-90% was obtained with the killed oral cholera vaccine containing the B-subunit but this level of protection declined to about 50% after 6 months. The modified killed (WC-OCV) lacking the B-subunit gave a somewhat lower initial level of protection but after 6 months the protection afforded by the two vaccines was similar [10, 11]. Unfortunately, the vaccine is prohibitively expensive for public health use in developing countries.

Starting in the mid-1980s, following technology transfer, Vietnamese scientists at the National Institute of Hygiene and Epidemiology (NIHE) in Hanoi developed and produced an oral, killed cholera vaccine for the country’s public health programs. A two-dose regimen of a first generation monovalent (anti-O1) cholera vaccine produced at US$ 0.10 per dose underwent a field trial in Hue, Vietnam [12]. The calculated efficacy against El Tor cholera was 66% in fully immunized adults and children. Subsequently, killed O139 whole cells were added to the Vietnamese vaccine due to the emergence of the new form of epidemic cholera caused by this serogroup. A study found the killed, bivalent WC-OCV to be safe and immunogenic in adults and children one year and older [13].

The Vietnamese vaccine has several distinct advantages over the Swedish vaccine. The Vietnamese vaccine confers protection against the El Tor biotype in younger children, less expensive making it feasible for public health programs in developing countries, while the Swedish vaccine is prohibitively expensive. Finally, it can be administered without a buffer, while the Swedish vaccine requires a buffer and stricter cold chain requirements.

Since licensure of the oral cholera vaccine in Vietnam, more than 9 million doses have been administered without any report of serious adverse events. The vaccine is produced according to recommended guidelines [13] at the Company for Vaccine and Biological Production No. 1 (VABIOTECH) in Hanoi. VABIOTECH is working towards WHO Good Manufacturing Practices (GMP) certification, which they hope to receive in the next few years. However, for the vaccine to be purchased by UN agencies it must be produced in a country with a WHO-recognized National Regulatory Agency (NRA) for which CENCOBI, the Vietnamese NRA is not. The vaccine was therefore reformulated; its production process modified and its production technology transferred to Shantha Biotechnics in India where the NRA, the Drugs Controller General of India (DCGI) is recognized by WHO.

Based on data from studies performed by the International Vaccine Institute (IVI), this modified killed WC-OCV was licensed in Vietnam to VaBiotech and in India to the Shantha Biotechnics in February 2009. Despite the recent licensure, there are remaining questions that need to be answered that would be vital in deploying the vaccine including optimization of dosing regimen. A previous study performed in Kolkata revealed that two doses of the vaccine when given 14 days apart did not result in higher immune response after the first dose [16], contrary to earlier findings with the Swedish vaccine [17, 18]. This new finding may be due to the higher lipopolysaccharide (LPS) content of the reformulated vaccine which may have elicited sufficient immune response that it effectively blocks subsequent antigen presentation with the second dose of the vaccine.

In order to assess if immune responses will be boosted if we prolong the interval between dosing of the modified killed WC-OCV, a Phase II double-blind, controlled, randomized trial to evaluate two different dosing intervals for the two-dose regimen will be performed in National Institute of Cholera and Enteric Diseases (NICED), Kolkata, India.

## Study Rationale

The absence of a boosting response after a 14 day interval with the two-dose regimen of the modified killed WC-OCV raises the possibility that a longer dosing interval may be required to observe a boost in the immune response. This study will compare the immune responses following 14-day and 28-day dosing intervals.

# STUDY OBJECTIVES

## Main Study

## 2.1.1 Primary objective:

To compare vibriocidal immune responses to the modified killed WC-OCV when given as two doses, 14 and 28 days apart, in healthy, non-pregnant adults and children volunteers.

**2.1.2 *Secondary objective:***

To confirm the safety of two doses of modified killed WC-OCV in healthy, non-pregnant adult and children volunteers

**2.2 *Additional Exploratory Study Objective:***

To explore feasibility of using other immunological indicators in determining the immunogenicity of modified killed WC-OCV in an additional cohort of 30 subjects:

(Fecal antibody to LPS, LPS-specific microELISPOT, and IgA to LPS endpoint ELISA)

# STUDY DESIGN

## Endpoints

### Primary Endpoints

Proportion of subjects exhibiting 4-fold or greater rises in titers of serum vibriocidal antibodies, relative to baseline, 14 days after last dose of study agent in each dose-interval group.

### Secondary Endpoints

1. Proportion of subjects exhibiting 4-fold or greater rises in titers of serum vibriocidal antibodies, relative to baseline, 14 days after first dose of study agent
2. Geometric mean serum vibriocidal titers at baseline, 14 days after each dose of the study agent, in each dose-interval group.
3. Proportion of subjects given 2 doses of study agent given 14 and 28 days apart with any of the following adverse events:
   1. Immediate reactions within 30 minutes after each dose
   2. Serious Adverse Events occurring throughout the trial
   3. Reactogenicity during three consecutive days following each dose: Headache, vomiting, nausea, abdominal pain/cramps, gas, diarrhea, fever, loss of appetite, general ill feeling
      1. Diarrhea is defined as having 3 or more loose/watery stools within a 24 hour period.
      2. Fever is defined as having an oral or tympanic temperature of ≥38^o^ C

**3.1.3 Additional Exploratory Study Endpoints:**

Proportion of subjects given 2 doses of vaccine given 14 and 28 days apart with significant responses to:

1. Fecal antibody to LPS
2. LPS-specific micro ELISPOT
3. IgA to LPS (by ELISA)

## Type of study

This study has two parts, both are randomized, double-blind, controlled trials. The first study is among healthy, non-pregnant adults (aged 18 years and above) and children (aged 1 – 17 years) randomized into one of following 2 arms:

| **Arm** | **Dose 1** | **Dose 2** | **Dose 3** |
| --- | --- | --- | --- |
| **1** | Bivalent killed  oral cholera vaccine | Bivalent killed  oral cholera vaccine | Killed *Escherichia coli* K12 placebo |
| **2** | Bivalent killed  oral cholera vaccine | Killed *Escherichia coli* K12 placebo | Bivalent killed  oral cholera vaccine |

The additional study explores other immune responses and includes only healthy, non-pregnant adults (aged 18 years and above) who will be randomized as above, but will have different phlebotomy schedules.

## Random Allocation

Eligible subjects will be assigned to receive the study agent according to randomization lists generated by the IVI. Three randomization lists will be generated by IVI. One randomization list will be generated for healthy children 1 to 17 years of age, one list will be generated for healthy, non-pregnant adults aged 18 years and above enrolled for evaluation of safety and optimal dosing interval, and one list will be generated for healthy, non-pregnant adults aged 18 years and above, enrolled for evaluation of safety and other immunological assays. The randomization lists will be generated by an IVI staff member who is otherwise not involved in the study. A copy of each list will be retained in the IVI. Study numbers will be assigned sequentially.

The randomization list will be sequential numbers unique to each individual. Randomization numbers will be generated in blocks of at least 4, which will include equal numbers of vaccines and placebos, to ensure that balance between treatments is maintained. Blocking will only be known to the statistician preparing the randomization list. Each randomization number will have a randomly pre-assigned group allocation (14 day or 28 day dosing interval).

Age-stratified randomization will be used for the children's study to ensure that each age group is represented, with approximately 59 to 60 children in each of the following age-groups: 1 to 5 years, 6 to 10 years, and 11 to 17 years. The randomization numbers generated in blocks of at least four will be followed sequentially as each child in enrolled.

## Blinding and Code Breaking Procedures

To protect blinding, study vaccines will be pre-labeled by the Shantha Biotechnics personnel who are not involved in the conduct or monitoring of the trial.

The IVI, safety monitor, and Shantha Biotechnics will hold the randomization list. Otherwise the list will not be revealed until the end of the trial and until the computerized data set to be used for the analysis has been frozen and a hard copy handed to the safety monitor.

Individual subject code-break envelopes will be provided to the investigator and held in a locked cabinet at the study site which is only accessible to authorized personnel. These envelopes will be available on a 24-hour basis and individual envelopes will only be opened in an emergency and where knowledge of the study agent received by the subject would impact on medical care provided. If any study agent assignment is un-blinded, safety monitor, IVI and Shantha Biotechnics will be notified immediately.

# RECRUITMENT AND ENROLMENT

The total target enrollment is 386 subjects, including 208 healthy, non-pregnant adults and 178 children.

The target enrollment for the main study population is 356 subjects, i.e. 178 adults and 178 children. 178 subjects receive one dose of placebo and two doses of vaccine with 14 days between vaccine doses and 178 subjects receive one dose of placebo and two doses of vaccine with 28 days between vaccine doses.

The target enrollment for assessment of other immunologic assays is 30 healthy, non-pregnant subjects. 15 subjects receive one dose of placebo and two doses of vaccine with 14 days between vaccine doses and 15 subjects receive one dose of placebo and two doses of vaccine with 28 days between vaccine doses.

## Recruitment

Residents of Kolkata not residing in the areas covered by the phase III trial will be recruited in the study. Recruitment will be performed in the NICED Clinical Trial Centre. Subjects will be instructed to proceed to the study center if they are interested in participating. Subjects will be recruited from the surrounding areas near the NICED where no clinical trial is currently being performed.

## Inclusion Criteria

Healthy, non-pregnant adults aged 18 years and above and healthy children aged 1 - 17 will be recruited in Kolkata.

All subjects must satisfy the following criteria at study entry:

1. Males or non-pregnant females aged 18 years and above and children aged 1 -17 years who the investigator believes will comply with the requirements of the protocol (i.e. available for follow-up visits and specimen collection).
2. Written informed consent obtained from the subjects or their parents/guardians, and written assent for children aged 12 – 17 years.
3. Healthy subjects as determined by:

- Medical history
- Physical examination
- Clinical judgment of the investigator

## Exclusion Criteria

The following criteria should be checked at the time of study entry, if any of the following is present then the subject will be excluded from the study:

1. Ongoing serious chronic disease
2. For females of reproductive age: Pregnancy (or females planning to become pregnant during the study period; as determined by verbal screening)
3. Immunocompromising condition or therapy (for corticosteroids this would mean ≥0.5 mg/kg/day)
4. Diarrhea (3 or more loose/watery stools within a 24-hour period) 6 weeks prior to enrollment
5. One or two episodes of diarrhea lasting for more than 2 weeks in the past 6 months
6. One or two episodes of abdominal pain lasting for more than 2 weeks in the past 6 months
7. Intake of any anti-diarrhea medicine in the past week
8. Abdominal pain or cramps, loss of appetite, nausea, general ill-feeling or vomiting in the past 24 hours
9. Acute disease one week prior to enrollment, with or without fever. Temperature ≥38ºC warrants deferral of the vaccination pending recovery of the subject
10. Receipt of immunoglobulin or any blood product during the past 3 months
11. Receipt of antibiotics in past 14 days
12. Receipt of live or killed enteric vaccine in past 4 weeks
13. Receipt of killed oral cholera vaccine

## Subject withdrawal during the study

Each subject is free to accept or reject the proposal to enroll in this study. Even after enrollment the subject will be able to withdraw from the study at any time.

The following criteria should be checked at each visit subsequent to the intake of the study agent:

1. Use of any immunosuppressive or immune-modifying drugs during the study period (for corticosteroids this would mean ≥0.5 mg/kg/day)

2. Administration of immunoglobulin or any blood product during the study period

If any of the above criteria is applicable, then it may affect the subject’s evaluation in the per-protocol analysis, however the individual will not be withdrawn from the study.

## Identification of subjects

An identification card will be provided to all study participants. Participants will be asked to bring the card whenever they return to the study center. Follow-up visit dates will be written in the identification cards.

As in earlier study, to ensure identification of subjects, we will use fingerprinting scanning in the verification of the identity of subjects [19]. This system was previously used in Vietnam [19] and Kolkata [16] and allowed accurate identification of subjects even when their cards were not available. However, if finger-prints are not identifiable for any subject of 1-3 years age then identification cards will also be used. Participants will be requested to provide a scanned image of a finger by resting it on a portable scanner. The fingerprint image will be linked to a unique identification number and stored in the biometrics database. Only the participant’s fingerprint template will be kept together with his/her identifiers (name, age, sex, address, or head of the household) as secondary keys for verification. For confidentiality, the template will be kept separate from his/her Case Report Form (CRF) data. Linkage will be made through each participant’s unique identification number and finger print scan. The linkage scheme for each participant is shown below:

Fingerprint

Unique identification number

Identifiers (name, age, sex, address, or head of the household)

Data from Case Report Forms

The fingerprint will be used for the purposes of this research only. All electronic data will be password-protected and only staff directly involved in the study will have access to this data. All paper copies of the data and electronic backup files will be kept in a secure cabinet and only staff directly involved in the study will have access to this data.

During subsequent contacts with the study staff (i.e. during follow-up visits and administration of the second dose of the study agent), participants will again be requested to provide a scanned image of his/her finger by resting it on a portable scanner. The fingerprint will be used to search and identify the participant’s unique identification number. The technology is such that only a few seconds are required to identify the participant so that this will not cause undue burden in terms of waiting time.

## Duration of the study period for one subject

Each study subjects’ participation will last for 42 days (up to 44 days).

# STUDY AGENTS

## Vaccine

The reformulated modified killed oral cholera vaccines are provided by the Shantha Biotechnics. Each vaccine dose contains the following:

| **Vaccine strain** | **Reformulated version** |
| --- | --- |
| *V. cholerae* O1 Inaba El Tor strain Phil 6973 formalin killed | 600 Elisa units (EU) of lipopolysaccharide (LPS) |
| *V. cholerae* O1 Ogawa classical strain Cairo 50 heat killed | 300 EU LPS |
| *V. cholerae* O1 Ogawa classical strain Cairo 50 formalin killed | 300 EU LPS |
| *V. cholerae* O1 Inaba classical strain Cairo 48 heat killed | 300 EU LPS |
| *V. cholerae* O139 strain 4260B formalin killed | 600 EU LPS |

Aliquots from the individual lots of cholera vaccine have undergone extensive quality control testing at Shantha Biotechnics for sterility, detoxifying agent, thiomersal assay, immunogenicity in animals, functional residual cholera toxin activity (rabbit skin test) and LPS content by an ELISA method using polyclonal antibody.

## Placebo

The placebo contains heat-killed *Escherichia coli* *(E. coli)* K12 strain in an amount whose optical turbidity is identical to that for the modified killed WC-OCV. *E.coli* K-12 was originally isolated from a convalescent diphtheria patient in 1922 [20]. ‘It lacks virulence characteristics, grows readily on common laboratory media and has proven to be a standard bacteriological strain used in microbiological research and teaching. The potential for *E.coli* K 12 strains to colonize the human colon is quite low so these are not likely to pose a risk to human or animal health, to plants, or to other microorganisms. These strains have been utilized for 70 years, often in industrial settings with high volumes and cell densities and also have been employed extensively in research laboratories. The *E. coli* K12 strain is considered highly safe for human handling and consumption and produces no adverse effects’ [21, 22].

The strain of *E. coli* K12 lot supplied by the Shantha Biotechnics Ltd. was originally obtained from the Korean Research Institute of Biotechnology and Biosciences. The master seed of this K12 strain was made at the Shantha Biotechnics as a frozen glycerol stock and characterized for its identity and purity. It was not found genetically modified. Using this master seed lot, *E. coli* K12 placebo was prepared by formaldehyde and heat inactivation under Good Manufacturing Practice conditions at the Shantha Biotechnics; and was adequately characterized for sterility, identity, thiomersal and formaldehyde content. Thus, it is highly safe for human handling and consumption and produces no adverse effects.

## Packaging and coding

The vaccine and placebo comes in single-dose vials containing 1.5 ml of study agent. All individual vials will have a blinded identification number/code on the vial based on the randomization list generated by the IVI. Vials will be labeled in Shantha Biotechnics, Hyderabad by staff that is not otherwise involved in the trial.

## Study agent accountability

Complete and accurate written records of receipt and storage and utilization of the study agent including: date received, lot number, quantity received and doses administered (with the identification of the subject) must be maintained by the site study staff. Any known discrepancies in the accountability of the study agent must be adequately documented. At the end of the trial, the unused study agent will be returned to Shantha Biotechnics by the investigator or disposed of according to the instructions provided by Shantha Biotechnics. The investigator will not use the study agent in any other manner than that provided for in the protocol.

The principal investigator will obtain the necessary clearances from the appropriate local agencies for use in clinical trials of the study agents. Shantha will obtain the necessary clearances from the Central Research Institute, Kasauli. The agents will be kept in a secure place.

## Storage conditions

The study agents will be stored in a secure place between 2º and 8ºC before administration.

## Administration

After acquisition of informed consent and ascertainment of eligibility, consenting, eligible subjects will be entered into the trial in the randomization sequence, as noted above.

Vaccine recipients will receive 1.5 ml of orally delivered modified, killed cholera vaccine per dose and placebo recipients will receive 1.5 ml of orally delivered *E. coli* K12 placebo.

At the time of the first dose, information about study agent administration will be entered into CRF Day 0. This information will note the randomization number, the success of administration as well as certain additional information.

Cups or needle-less syringes used for vaccination with the study agent will be disposed of; after each dose, to prevent inadvertent administration of contaminating amounts of non-assigned agents.

Fourteen days (and up to 16 days) after the first dose, a second dose will be administered and 28 days (and up to 30 days) after the first dose, a third dose will be administered, according to the same procedures.

Contraindications to the second dose or third dose will be:

- 1. The occurrence, after the first dose or second dose, of a severe allergic reaction (generalized urticaria, wheezing, anaphylaxis)
  2. The development of any illness after the first dose or second dose, judged by the Principal Investigator to be too severe to continue participation
  3. A diagnosis of pregnancy after the first dose or after the second dose through verbal screening (irregularity of menses)

Indications warranting deferral of the second dose or third dose, pending recovery of the subject will be:

- 1. Any acute disease, with or without fever, within the past 48 hours
  2. Loose or watery stools (with or without blood), abdominal pain or cramps, loss or appetite, nausea, general ill feeling, fever or vomiting within the past 24 hours.

# STUDY PROCEDURES

## Schedule and description of observations and visits

*See flowcharts on page vii.*

For 356 subjects in main study population (Groups A and B):

1. **Day 0**: Informed consent is obtained from the subject or parent/guardian. If the subject is 12-17 years old, then assent is also obtained after the informed consent is signed (or marked with thumbprint) by the parent/guardian. A fingerprint template is obtained at this time for identification. Next, screening for inclusion and exclusion criteria, history and physical examination are completed by the study physicians. Then approximately 3.5 to 5 ml of blood is obtained for baseline (pre-immunization) immunologic tests and the subject is randomized. Subjects are randomized following the randomization list as previously described in section 3.3. Subjects are logged into the randomization list in a sequential manner. The study agent is given according to the assigned randomization number. The subjects are asked to wait in the clinic for 30 minutes for adverse event monitoring. CRF Day 0 is completed.
2. **Day 1-3:** Subjects are followed-up for interval solicited adverse event monitoring (either they return to the center or are visited at home). CRF Day 1, Day 2 and Day 3 are completed.
3. **Day 14 (*up to 16 days after administration of dose 1*)**: Subjects return to the study center for screening, interval clinical evaluation and adverse event monitoring. Identity is confirmed by fingerprinting. Approximately 3.5 to 5 ml of blood is obtained for testing of immunologic response. The second dose of study agent is given according to the assigned randomization number. The subject is asked to wait in the clinic for 30 minutes for adverse event monitoring. CRF Day 14 is completed.
4. **Day 15-17**: Subjects are followed-up for interval solicited adverse event monitoring (either they return to the center or are visited at home). CRF Day 15, Day 16 and Day 17 are completed.
5. **Days 28 (*up to 30 days after administration of dose 1*)**: Subjects return to the study center for screening, interval clinical evaluation and adverse event monitoring. Identity is confirmed by fingerprinting. Approximately 3.5 to 5 ml of blood is obtained for testing of immunologic response. The third dose of study agent is given according to the assigned randomization number. The subject is asked to wait in the clinic for 30 minutes for adverse event monitoring. CRF Day 28 is completed.
6. **Day 29-31**: Subjects are followed-up for interval solicited adverse event monitoring (either they return to the center or are visited at home). CRF Day 29, Day 30 and Day 31 are completed.
7. **Day 42 (up to 44 days after administration of dose 1)**: Subjects return to the study center for interval clinical evaluation including adverse event monitoring. Identity is confirmed by fingerprinting. Approximately 3.5 to 5 ml of blood is obtained for testing of immunologic response. CRF Day 42 and the Study Summary are completed.

For the additional cohort of 30 subjects for assessment of other immunologic assays (Group C):

1. **Day 0**: Informed consent is obtained from the subject. A fingerprint template is obtained at this time for identification. Next, screening for inclusion and exclusion criteria, history and physical examination are completed by the study physicians. A stool sample is collected for fecal antibody assay followed by collection of approximately 5 ml of blood for baseline (pre-immunization) immunologic tests. The stool sample in subsequent visits is collected either at the trial centre or they will be asked to collected stool samples at their home in a container provided to them beforehand. Subjects are then randomized following the randomization list as previously described in section 3.3. Subjects are logged into the randomization list in a sequential manner. The study agent is given according to the assigned randomization number. The subjects are asked to wait in the clinic for 30 minutes for adverse event monitoring. CRF Day 0 is completed.
2. **Day 1-3:** Subjects are followed-up for interval solicited adverse event monitoring (either they return to the center or are visited at home). CRF Day 1, Day 2 and Day 3 are completed.
3. **Day 7**: Subjects return to the study center for adverse event monitoring and sample collection. Identity is confirmed by fingerprinting. A stool sample is collected for fecal antibody assay and approximately 5 ml of blood is obtained for testing of immunologic response. CRF Day 7 is completed.
4. **Day 14 (*up to 16 days after administration of dose 1*)**: Subjects return to the study center for screening, interval clinical evaluation and adverse event monitoring. Identity is confirmed by fingerprinting. A stool sample is collected for fecal antibody assay. The second dose of study agent is given according to the assigned randomization number. The subject is asked to wait in the clinic for 30 minutes for adverse event monitoring. CRF Day 14 is completed.
5. **Day 15-17**: Subjects are followed-up for interval solicited adverse event monitoring (either they return to the center or are visited at home). CRF Day 15, Day 16 and Day 17 are completed.
6. **Day 21**: Subjects return to the study center with identity confirmed by fingerprinting. A stool sample is collected for fecal antibody assay and approximately 5 ml of blood is obtained for immunologic tests. CRF Day 21 is completed.
7. **Days 28 (*up to 30 days after administration of dose 1*)**: Subjects return to the study center for screening, interval clinical evaluation and adverse event monitoring. Identity is confirmed by fingerprinting. A stool sample is collected for fecal antibody assay. The third dose of study agent is given according to the assigned randomization number. The subject is asked to wait in the clinic for 30 minutes for adverse event monitoring. CRF Day 28 is completed.
8. **Day 29-31**: Subjects are followed-up for interval solicited adverse event monitoring (either they return to the center or are visited at home). CRF Day 29, Day 30 and Day 31 are completed.
9. **Day 35**: Subjects return to the study center with identity confirmed by fingerprinting. A stool sample is collected for fecal antibody assay and approximately 5 ml of blood is obtained for immunologic tests. CRF Day 35 is completed.
10. **Day 42 (up to 44 days after administration of dose 1)**: Subjects return to the study center for interval clinical evaluation including adverse event monitoring. Identity is confirmed by fingerprinting. A stool sample is collected for fecal antibody assay. CRF Day 42 and the Study Summary are completed.

## Assessment after vaccination

### Follow-up for Adverse Events

Following each dose, subjects will be observed in the clinic (vaccinating area) for 30 minutes to assess for any immediate reactions.

After each dose of the study agent, subjects will be followed up on an out-patient basis (either asked to return to the center or followed up at home) for three days. The subject will provide a 24-hour recall history of symptoms and temperature will be taken. Study staff will complete the appropriate CRF pages. Any unsolicited adverse events will be documented on CRF Appendix for Adverse Events.

In addition to the three days of follow up following each dose, interval clinical evaluation will be performed on Day 14, 28 and 42 to assess for any adverse events that may have occurred. Any adverse events noted will be recorded on CRF Appendix for Adverse Events.

Any medications taken, non- solicited Adverse Events and Serious Adverse Events reported during the study period will be recorded on the appropriate Appendix pages of the CRF.

The current standard procedures of the NICED for the management and reporting of Adverse Events Following Immunization (AEFI) will be followed in the event of any AEFI during this study.

### Follow-up for Serious Adverse Events

Any serious adverse event which occurs during a subject’s participation in the study will be reported using the Serious Adverse Events Form in the Appendix pages of the CRF.

A serious adverse event (experience) is any untoward medical occurrence that at any dose:

- Results in death,
- Is life-threatening. The term “life-threatening” in the definition of “serious” refers to an event in which the subject was at risk of death at the time of the event; it does not refer to an event, which hypothetically might have caused death, if it were more severe.
- Requires in subject hospitalization or prolongation of existing hospitalization,
- Results in persistent or significant disability/incapacity,
- Results in a congenital anomaly/birth defect, or
- Any other important medical event that may not be immediately life-threatening or result in death or hospitalization but may jeopardize the subject or may require medical or surgical intervention to prevent one of the other outcomes listed in the definition above. Prudent medical judgment must be exercised to decide whether reporting is appropriate. An example includes treatment for allergic bronchospasm that does not result in hospitalization but required intensive medical intervention in the emergency room.

Medical and scientific judgment should be exercised in deciding whether expedited reporting is appropriate in other situations, such as important medical events that may not be immediately life-threatening or result in death or hospitalization but may jeopardize the subject or may require intervention to prevent one of the other outcomes listed in the definition above. These should also usually be considered serious.

### Blood Draws

For 356 subjects in main study group:

Venipuncture to draw approximately 3.5 to 5 ml blood will be performed prior to dose 1, 14 days after dose 1, 14 days after dose 2 and 14 days after dose 3 for the 356 subjects in the main study group. Each specimen will be labeled with the following information: date of blood draw, initials, and study ID number.

At the time of the first bleed CRF Day 0 will be completed to indicate the success of the blood collection. At the time of second bleed CRF Day 14 will be completed, at the time of the third bleed CRF Day 28 will be completed, and at the time of the forth bleed CRF Day 42 will be completed in a similar manner. During all the blood draws, numbered laboratory stickers will be affixed to the corresponding CRF and specimen.

For 30 subjects in the subset for assessment of other immunologic assays:

Venipuncture to draw 5 ml blood; will be performed prior to dose 1, 7 days after dose 1, 7 days after dose 2 and 7 days after dose 3 for the 30 subjects in the subset for assessment of other immunologic assays. Each specimen will be labeled with the following information: date of blood draw, initials, and study ID number.

At the time of the first bleed CRF Day 0 will be completed to indicate the success of the blood collection. At the time of second bleed CRF Day 7 will be completed, at the time of the third bleed CRF Day 21 will be completed, and at the time of the forth bleed CRF Day 35 will be completed in a similar manner. During all the blood draws, numbered laboratory stickers will be affixed to the corresponding CRF and specimen.

### Stool Samples

For the additional cohort of 30 subjects for assessment of other immunologic assays only:

Stool samples will be collected prior to dose 1, 7 and/or 14 days after dose 1, 7 and/or 14 days after dose 2 and 7 and/or 14 days after dose 3 from the additional cohort of 30 adult for assessment of other immunologic assays. The stool sample in subsequent visits is collected either at the trial centre or they will be asked to collected stool samples at their home in a container provided to them beforehand. Each specimen will be labeled with the following information: date of stool collection, subject initials and study ID number.

CRF Day 1, 7, 14, 21, 28, 35 and 42 will be completed to indicate whether each stool sample was provided. For all stool samples numbered laboratory stickers will be affixed to the corresponding CRF and specimen.

### Sample Storage

After study testing, and in accordance with subject instruction obtained during informed consent, remaining samples may be destroyed or stored for future use. On agreement from the PI and IVI coordinator all remaining samples may also be destroyed. All samples confirmed for long-term storage will be securely held at the IVI. Stored samples will retain original study labels with no personal identifiers.

## Laboratory procedures

#### *Serum vibriocidal antibody assay*

The vibriocidal antibody assay is a bactericidal assay requiring the presence of complement-fixing antibody bound specifically to vibrios; this serum antibody response increases after clinical cholera or after vaccination. The serum samples from the volunteer prior to immunization and 14 days after each dose will be tested using vibriocidal antibody assay. An increase of titer by 4-fold or greater between baseline and post-immunization sera will be considered a significant antibody response. The vibriocidal assay using the microtiter technique will be performed in the NICED.

#### *Fecal antibody assay*

Antibody excreted into feces will be measured to assess mucosal immune response. Stool samples collected from volunteers prior to immunization and 7 and/or 14 days after each dose will be tested using the fecal antibody assay. Secretory IgA titers will be determined by ELISA. The fecal antibody assay will be performed at the NICED.

#### *LPS-specific microELISPOT*

The Enzyme-linked Immunosorbent spot (ELISPOT) assay will be used to measure type and quantity of antibody secreting cells. Blood collected from subjects prior to immunization, and 7 days after each dose will be tested using the microELISPOT assay. The microELISPOT assay will be performed at the NICED.

#### *IgA to LPS by ELISA*

Plasma samples will be assayed for measurement of LPS-specific antibody titers. Blood collected from subjects prior to immunization, and 7 days after each dose will be tested by the ELISA method. The ELISA assay will be performed at NICED.

# ADVERSE EVENTS

## Definitions

ADVERSE EVENTS:

An adverse event is defined as any noxious, pathologic, or unintended change in anatomic, physiologic, or metabolic functions, as indicated by physical signs, symptoms, and/or laboratory changes occurring in any phase of the clinical trial, regardless of their relationship to study medication. Adverse events include:

- an exacerbation of a pre-existing condition
- any illness happening in between the two doses’ administration period
- any drug interaction
- any event related to a concomitant medication
- pregnancy

A treatment-emergent event is defined as any event not present prior to exposure to study medication or any event already present that worsens in either intensity or frequency following exposure to study medication.

Possible adverse events would include abdominal pain, loss of appetite, nausea, general ill feeling, fever and vomiting. Follow-up for adverse events following immunization will be conducted and recorded as described in section 6.2.

SERIOUS ADVERSE EVENTS:

A Serious Adverse Event means any event that results in:

- death
- is immediately life-threatening
- results in persistent or significant disability/incapacity
- requires inpatient hospitalization or prolongation of existing hospitalization
- is a congenital anomaly/birth defect
- Any other medically important condition that required intervention to prevent one of the above criteria.

Important medical events that may not result in death, be life-threatening, or require hospitalization may be considered a serious adverse event when, based upon appropriate medical judgment, they may jeopardize the subject and may require medical or surgical intervention to prevent one of the outcomes listed in this definition. Examples of such medical events include allergic bronchospasm requiring intensive treatment in an emergency room or at home, blood dyscrasias or convulsions that do not result in in-subject hospitalization, or the development of drug dependency or drug abuse.

## Assessment of Causality

The Investigator’s assessment of an adverse event’s relationship to study drug is part of the documentation process, but it is not a factor in determining what is or is not reported in the study. If there is any doubt as to whether a clinical observation is a treatment-emergent adverse event, the event should be reported. The relationship of administration of the study agent to the serious adverse event will be assessed as follows:

Very Likely/Certain: A clinical event with a plausible time relationship to vaccine administration and which cannot be explained by concurrent disease or other drugs or chemicals.

Probable: A clinical event with a reasonable time relationship to vaccine administration; is unlikely to be attributed to concurrent disease or other drugs or chemicals.

Possible: A clinical event with a reasonable time relationship to vaccine administration, but which could also be explained by concurrent disease or other drugs or chemicals.

Unlikely: A clinical event whose time relationship to vaccine administration makes a causal connection improbable, but which could be plausibly explained by underlying disease or other drugs or chemicals

Unrelated: A clinical event with an incompatible time relationship and which could be explained by underlying disease or other drugs or chemicals.

Unclassifiable: A clinical event with insufficient information to permit assessment and identification of the cause.

## Assessment of Severity

The intensity of the adverse event will be rated adapting the guidelines, where applicable, set by the U.S. FDA Toxicity Grading Scale for Healthy Adult and Adolescent Volunteers Enrolled in Preventive Vaccine Clinical Trials [23] except for mild diarrhea, which will be defined as 3 loose or liquid stools in a 24 hour period.

| **Systemic  (General)** | **Mild  (Grade 1)** | **Moderate  (Grade 2)** | **Severe  (Grade 3)** | **Potentially Life Threatening (Grade 4)** |
| --- | --- | --- | --- | --- |
| Nausea/vomiting | No interference with activity or 1 - 2 episodes/24 hours | Some interference with activity or > 2 episodes/24 hours | Prevents daily activity, requires outpatient IV hydration | ER visit or hospitalization for hypotensive shock |
| Diarrhea | 3 loose stools or < 400 gms/24 hours | 4 - 5 stools or 400 - 800 gms/24 hours | 6 or more watery stools or > 800gms/24 hours or requires outpatient IV hydration | ER visit or hospitalization |
| Headache | No interference with activity | Repeated use of non-narcotic pain reliever > 24 hours or some interference with activity | Significant; any use of narcotic pain reliever or prevents daily activity | ER visit or hospitalization |
| Fatigue | No interference with activity | Some interference with activity | Significant; prevents daily activity | ER visit or hospitalization |

| **Systemic Illness** | **Mild  (Grade 1)** | **Moderate  (Grade 2)** | **Severe  (Grade 3)** | **Potentially Life Threatening (Grade 4)** |
| --- | --- | --- | --- | --- |
| Illness or clinical adverse event (as defined according to applicable regulations) | No interference with activity | Some interference with activity not requiring medical intervention | Prevents daily activity and requires medical intervention | ER visit or hospitalization |

Changes in the severity of an adverse event should be documented to allow an assessment of the duration of the event at each level of intensity to be performed. Adverse events characterized as intermittent require documentation of onset and duration of each episode.

## Reporting of Serious Adverse Events

Serious adverse events will be reported promptly to the on-site safety monitor once the principal investigator or designee determines that the event meets the protocol definition of a SAE. The safety monitor will provide an assessment of causality.

- The investigator or designee will fax the SAE report to the IVI (through the external monitor), and to the Shantha Biotechnics (through the medical monitor, Dr Mandeep Singh Dhingra) within 24 hours of his/her becoming aware of these events.
- The SAE form will always be completed as thoroughly as possible with all available details of the event, assessment of causality, and signed by the investigator (or designee).  If the investigator does not have all information regarding an SAE, he/she will not wait to receive additional information before notifying the IVI, and Shantha Biotechnics.
- After the initial SAE report, the investigator is required to proactively follow each subject and provide further information to the IVI and Shantha Biotechnics on the subject’s condition. The investigator (or designee) will follow-up subjects with SAEs until the event has: resolved, subsided, stabilized, or disappeared or the event is otherwise explained, or the subject is lost to follow-up.
- The date of final disappearance of the adverse event will be documented.
- The safety monitor will always provide an assessment of causality at the time of the initial report. The Medical Monitor may request that the investigator perform or arrange for the conduct of supplemental measurements and/or evaluations to elucidate as fully as possible the nature and/or causality of the serious adverse event. The investigator is obliged to assist.
- All SAEs will be reported to the NICED-IEC and the IVI-IRB according to their respective rules and regulations

# DATA COLLECTION AND MANAGEMENT PROCEDURES

## Case Report Forms (CRF)

Electronic Case report forms (CRF) will be used in this study. Electronic CRFs and workbooks will be maintained for recording data for each subject enrolled in the study. The investigator is responsible to ensure the accuracy, completeness, legibility and timeliness of the data reported to the sponsor in the electronic CRFs and workbooks. Data reported in the electronic CRFs derived from the workbooks and source documents should be consistent with the workbooks and source documents or the discrepancies should be explained. IVI will provide guidance to investigators on making corrections to the electronic CRF and workbooks.

## Source Documentation

Each subject requires complete and adequate source documentation (hospital or medical records, lab reports, test results) for the complete period of the study, unless the data recorded directly into the electronic CRF is considered the source data. These records must be available to IVI and regulatory authorities upon request for review.

## Data Management

Study personnel will extract all data collected in source documents and workbooks for computerization into the electronic CRF. Data will be double-entered into computers in a dedicated area located at the study site, using data entry programs specially created for the project by the IVI.

These programs will utilize custom-made software; all programs will incorporate identification of the keypunching errors, range and consistency checks *pari passu* with data entry. This software will provide error reports, exception lists, and summary reports for each activity. The software will also automatically back-up data at systematic intervals onto local hard disks and external medias, and will provide for an audit trail of all sequential changes made.

Data security for this data management system will be augmented by automatic computer virus scanning at start-up of each data entry and data management session, and password protection for accessing data and data management software. In addition, backup files generated by the data management software will be kept in a secure cabinet.

##### Data entry and cleaning will be conducted onsite and the IVI will be the data coordinating center. Final data cleaning, data freezing and data analysis will be performed at the IVI. All data management will be undertaken without access to the randomization code of study subjects.

# STATISTICAL CONSIDERATIONS

## Sample Size Calculation

Sample size was driven by seroconversion after two doses under 14 day and 28 day dosing intervals. We assumed there was no difference in seroconversion after two doses under a 14 day dosing interval compared to a 28 day dosing interval.

Among adult subjects we assumed 45% seroconversion after 2 doses and wished to exclude a >20% higher serconversion rate under a 28 day dosing interval with one-tailed α =0.05 and 80% power. Assuming a 15% drop-out rate, and using the method of Farrington and Manning for precision-based sample size calculations [24], a total of 89 subjects per study group would be needed.

Among child subjects we assumed 80% seroconversion after 2 doses and wished to exclude a >16% higher seroconversion rate under a 28 day dosing interval with one-tailed α =0.05 and 80% power. Assuming a 10% drop-out rate, and using the method of Farrington and Manning for precision-based sample size calculations [24], a total of 89 subjects per study group would be needed.

## Analysis Plans

Both intention-to-vaccinate and per protocol analysis will be performed.

*Intention-to-Vaccinate Analysis*

Every subject randomized in the study (who receives the correct or incorrect study agent, complete or incomplete doses) will be analyzed for safety, except if he/she did not receive any dose of the study agent (vaccine or placebo).

*Per-Protocol Analysis*

A per protocol analysis will compare subjects according to the study agent actually received and will include only those subjects who satisfied the inclusion/exclusion criteria, followed the protocol, completed all visits and received the correct dose. The following non-compliant subjects will be excluded:

- Subjects included without meeting at least one inclusion criterion
- Subjects included despite meeting at least one exclusion criterion
- Subjects found non compliant with the blood sampling schedule.
- Subjects vaccinated with the wrong study agent (non compliance with the randomization code)
- Subjects found non compliant with the study agent dosing schedule (non compliance with number of doses or non compliance with assigned dosing interval)
- Subjects excluded from the intention to vaccinate analysis.

*Analysis of demographics*

Demographic characteristics of subjects enrolled will be tabulated by group and overall.

*Analysis of safety*

Any adverse event that occurs prior to randomization will not be included in safety analysis. The number and percentage of subjects (with 95% CI) with diarrheal adverse event will be compared. In addition, the number and percentage of subjects (with 95% CI) with at least one adverse event (solicited and/or unsolicited) after vaccination and during the 6 weeks follow up period will be compared between the study groups.

The number and percentage of subjects with at least one Serious Adverse Event, with the frequencies of each type of event will be compared between the study groups.

Over-all rates of adverse reaction will be analyzed using the chi-square test or by the Fisher’s exact test when the numbers are sparse.

*Analysis of immunogenicity (vibriocidal immune response)*

Demonstration of a four-fold or greater rise in serum anti-O1 vibriocidal antibody titer will be the primary measure of vaccine immunogenicity. Geometric mean fold rises of serum titers will also be analyzed and compared. The number and percentage of subjects (with 95% CI) who exhibit at least a fourfold rise in serum anti-O1 vibriocidal titer after vaccination will be compared between the study groups.

Serum vibriocidal titers and fold-rises may be logarithmically transformed prior to statistical analyses in order to better approximate normality. Student’s t-test will be performed for continuous outcomes. Seroconversion will be compared using the chi-square test with Yates correction or by the Fisher’s exact test if the numbers are sparse. Analysis of covariance may be used to adjust for imbalances in baseline titers.

*Interim analysis*

No interim analysis is planned.

# MONITORING, AUDITING, INSPECTION

## Responsibilities of the investigator(s)

The site principal investigator will conduct the study in accordance with this protocol and will attempt to recruit the required number of patients in a reasonable period of time so as to complete the trial at the earliest. The site principal investigator will provide copies of the protocol to all the members of his study team. He or she will discuss this material with them and conduct training to assure that all the members of their study team are fully informed regarding the study agents and the conduct of the study. He or she will ensure that all his associates, colleagues and employees assisting in the conduct of this study are informed about their obligations in meeting their respective commitments. The principal investigator will provide a final report of the study.

The site principal investigator will ensure that all case report forms will be completed and computerized in real time (that is within 24 to 48 hours of completion of the form) to assure accurate and timely data. Any forms with queries or inconsistencies noted during data entry will be sent back to the field for correction or clarification.

## Responsibilities of the IVI coordinators

The coordinators from IVI will ensure that the trial is adequately monitored. At regular intervals, contact with the study site will be made through visits, e-mail, and telephone calls to review the study progress, adherence to the protocol, and any problems. During the monitoring visits, the following will be examined: subject informed consent, subject recruitment and follow-up, study agent allocation, study agent storage and transport, follow-up of subjects, and laboratory procedures. The IVI coordinators will discuss any problems with the investigators and define, after deliberation, any action(s) to be taken.

## Responsibilities of the Safety Monitor

The safety monitor will be responsible for overseeing any adverse events among the subjects. The safety monitor is independent of the investigators team and will be able to monitor subject consent/assent forms, case report forms and the severe adverse events report forms; at frequent intervals.

This study will not be having any Data Safety Monitoring Board for the following reasons: 1. The vaccine to be used for this study is already licensed in India. 2. No interim analysis will be made. 3. We do not foresee any greater risk from this study as we shall be using the same vaccine and the community where we plan to recruit has similar characteristics as the previous communities where previous studies on immune responses following one and two doses (16), phase II study were conducted; and Phase III currently being conducted by the same site investigators. 4. The only difference in conduct of this study is extended interval of 28 days between the first and second dose with an objective to measure any booster immune response after the second dose.

# ETHICAL AND REGULATORY STANDARDS

## Ethical principles/ Laws and regulations

The study will be conducted in compliance with the procedures outlined in this protocol, the International Conference on Harmonization’s Good Clinical Practice Guidelines (ICH-GCP) and in accordance with the ethical guidelines and local regulatory requirements for the trial. The investigators’ responsibilities will follow the WHO guidelines for GCP. It is also expected that local ethics committees will follow guidelines set forth by the WHO to ensure quality of the ethical review.

Electronic data will be password protected with additional securities as described in Section 8 above. Paper records and other non-electronic information collected during the study will be kept in locked cabinets under supervision with access limited to authorized study staff and investigators.

## Potential risks and risk minimization

The justification for using a placebo in this study is that a valid assessment of vaccine immunogenicity under two alternative dosing schedules can only be obtained with use of a placebo. The placebo will consist of heat-killed bacteria (*E.coli*), which belongs to the normal human gastro-intestinal flora and produces no adverse effects [20, 21, 22].

Risk-benefit. This formulation of the modified bivalent killed WC-OCV has not been reported to be associated with major adverse reactions in the course of the phase III trial currently being performed in Kolkata. Though this vaccine has not been tested specifically in pregnant women but the results from the use of its earlier version have indicated that there are likely to be no foreseeable risks to reproduction or to the fetus. Still, the pregnant volunteering women or those planning to become pregnant during the study period will be excluded from this study. The potential benefits to participants are substantial, since cholera is endemic in many parts of India. Evaluation of alternative dosing schedules will result in optimal use of this vaccine in India and endemic countries worldwide.

Benefit to all participants. All subjects receive two doses of vaccine. Knowledge regarding the use of this vaccine in a population with endemic cholera will be useful for future use of this vaccine in India. No pro-rated payment will be given, but reimbursement for transportation expenses and time lost from work will be given. Vitamins or food will be offered after each blood sample is taken.

## Informed consent and Assent

In obtaining and documenting informed consent and assent, the investigator must comply with the applicable regulatory requirements, GCP guidelines and ethical principles. The written informed consent form and assent form must be approved by and Institutional Review Board/Ethics Committee (IRB/EC) prior to its use.

The written informed consent will be obtained prior to enrolment for all participants. Subjects, or their parents and/or guardians (if subject is less than 18 years of age) will read the informed consent, and be allowed to ask questions regarding the study. If the subject or parents/guardians cannot read, the informed consent will be read and explained to them. The subject or their parent/guardian must sign (or thumb mark or “X” will be placed, if illiterate) and date the informed consent form prior to participating in any study-related activity. A literate witness must sign the informed consent form if the subject or parent/guardian is illiterate. The informed consent form must be signed and dated by the study personnel who obtained the consent.

A written assent form will be obtained prior to enrolment for all participants aged 12 – 17 years. They will read the assent form, and be allowed to ask questions regarding the study. If the subject cannot read, the assent will be read and explained to them. The subject will sign (or thumb mark or “X” will be placed, if illiterate) and date the form prior to any study-related activity. A witness must sign the assent form if the subject is illiterate. In addition, the assent form must be signed and dated by the study personnel who obtained the assent.

If information becomes available that may be relevant to the subject’s willingness to continue participating in the study, the investigator will inform the study subjects in a timely manner and a revised written informed consent must be obtained.

## Institutional Review Committee (IRB) / Ethics Committee (EC)

Before initiation of the study, the final protocol, and appropriate documents (information to be given to the subjects, informed consent forms, subject recruitment procedures, if any, investigator’s brochure, information sheets and advertisements) will be submitted to the IRB/EC of NICED, and IVI by the investigators. A copy of the study approval (including the informed consent approval) is to be kept in the Investigator’s study document binder and a copy is to be supplied to the IVI. Clearances from the appropriate local ethical review boards and the IVI Institutional Review Board will be obtained.

During the study, the investigator is responsible for providing the IRB/EC with all the documents subject to review (i.e. Protocol amendments, Informed consent updates, advertisements, and other written information to be provided to the subject). Appropriate reports on the progress and termination of the study will be made to the IRB/EC by the investigator in accordance with the IRB/EC guidelines and government regulations (as applicable)

# ADMINISTRATIVE ASPECTS

## Record retention

The investigator will retain trial related documents as required by the applicable regulatory requirement (s) or by an agreement with the sponsor. The investigator should take measure to prevent accidental or premature destruction of these documents.

Essential documents should be retained for:

- A period of two years after approval of a marketing application in an ICH region and until there is no pending or contemplated marketing applications in an ICH region.

OR

- A period of two years has elapsed since the formal discontinuation of clinical development of the investigational product.

The essential documents could be retained for a longer period however, if required by applicable regulatory requirements or by an agreement with the sponsor. It is the responsibility of the IVI and Shantha Biotechnics to inform the investigator as to when these documents no longer need to be retained.

## Publications

IVI, NICED, and Shantha Biotechnics shall jointly own the rights to the data, clinical, and biological specimens, results and other findings resulting from this trial. Parties are encouraged to publish the results of their work in a collaborative fashion for the benefit of the public while taking care to protect the intellectual property rights to proprietary discoveries. There shall be joint access of data. Guidelines for authorship of major, international, peer-reviewed journals will be used to establish authorship. Each party shall provide the others with a copy of each manuscript and abstract at least 30 days before submission for publication in a journal or presentation at an international meeting. The parties will have the right to examine the publication before it is printed and disseminated, and to request changes to the use of their name.

Subject to agreement on a case-by-case basis, each party is encouraged to produce and disseminate electronic versions of important publications produced as a result of this Cooperative Agreement. Each party will permit the others to disseminate such electronic versions as long as all original formatting, credits, and contents are maintained.

The contribution of all parties involved in this Cooperative Agreement shall be acknowledged in all abstracts, reports, or other peer-reviewed scientific publications containing data or information collected during the Project duration.

## Study Funding

This trial is part of a grant to the International Vaccine Institute by the Bill and Melinda Gates Foundation.

# REFERENCES

1. World Health Organization. Cholera, 2006*.* Wkly Epidemiol Rec, 2007; 82(31): p. 273-284.
2. World Health Organization. State of the art of new vaccines: research and development. Revised 2005, Geneva, Switzerland. WHO/IVB/05.
3. Zuckerman, JN, Rombo L, Fisch A. The true burden and risk of cholera: implications for prevention and control. *Lancet* 2007; 7:521-30
4. Government of India. Health Information of India 1995, DGHS, New Delhi
5. Sur D, Dutta P, Nair GB, Bhattacharya SK. Severe cholera outbreak following floods in a northern district of West Bengal. *Indian J Med Res* 2000; 112: 178-82.
6. Radhakutty G, Sircar BK, Mondal SK, Mukhopadhyay AK, Mitra RK, Basu A, Ichpugani I, Nair GB, Bhattacharya SK. Investigation of an outbreak of cholera in Allepey and Palghat districts, South India. *Indian J Med Res* 1997; 106: 455-7.
7. Niyogi SK, Mondal S, Sarkar BL, Garg S, Banerjee D, Dey GN. Outbreak of cholera due to *V cholerae* O1 in Orissa state. *Indian J Med Res* 1994; 100: 217-8.
8. Ramarmurthy P, Garg S, Sharma R, Bhattacharya SK, Nair GB, Shimada T,, Karasawa T, Kurazono H, Pal A, Takeda Y. Emergence of a novel strain of *Vibrio cholerae* with epidemic potential in southern and eastern India. *The Lancet* 1993; 341: 703-4.
9. World Health Organization. Meeting of the Strategic Advisory Group of Experts on immunization, October 2009: conclusions and recommendations. Wkly Epidemiol Rec 2009; 84(50):517-532.
10. Clemens J D et al , Field trials of cholera vaccines in Bangladesh: results from a three year follow- up. *The Lancet* 1990; 335: 270 –3.
11. Sanchez J L, Vasquez B, Beque R et al, Protective efficacy of the oral whole cell / recombinant B subunit cholera vaccine in Peruvian military recruits. *The Lancet* 1994; 344: 1273 – 6.
12. [Trach DD, Clemens JD, Ke NT, Thuy HT, Son ND, Canh DG, Hang PV, Rao MR.](http://www.ncbi.nlm.nih.gov:80/entrez/query.fcgi?cmd=Retrieve&db=PubMed&list_uids=9014909&dopt=Abstract) Field trial of a locally produced, killed, oral cholera vaccine in Vietnam. *The* *Lancet* 1997; 349, 231-5.
13. [Trach DD, Cam PD, Ke NT, Rao MR, Dinh D, Hang PV, Hung NV, Canh DG, Thiem VD, Naficy A, Ivanoff B, Svennerholm AM, Holmgren J, Clemens JD.](http://www.ncbi.nlm.nih.gov:80/entrez/query.fcgi?cmd=Retrieve&db=PubMed&list_uids=11884967&dopt=Abstract) Investigations into the safety and immunogenicity of a killed oral cholera vaccine developed in Viet Nam. *Bulletin of the World Health Organization* 2002; 8, 2-8.
14. Anh DD, Lopez AL, Canh DG, Thiem VD, Sonh NN, et al. Safety and Immunogenicity of the reformulated Vietnamese bivalent killed, whole cell oral cholera vaccine in adults. *Vaccine* 2007; 25: 1149-1155.
15. Mahalanabis D, Lopez AL, Sur D, Deen J, Manna B, et.al. A randomized, placebo-controlled trial of the bivalent killed, whole-cell, oral cholera vaccine in adults and children in a cholera endemic area in Kolkata, India. *PLoS ONE* 2008: 3 (6): e2323. doi:10.1371/journal.pone.0002323.

16. Kanungo S, Paisley A, Lopez AL, Bhattacharya M, Manna B, Kim DR, et al. Immune responses following one and two doses of the reformulated, bivalent, killed, whole-cell, oral cholera vaccine among adults and children in Kolkata, India: a randomized, placebo-controlled trial. *Vaccine* 2009; 27(49):6887-93.

17. Clemens JD, Stanton BF, Chakraborty J, Sack DA, Khan MR, Huda S, et al. B subunit-whole cell and whole cell-only oral vaccines against cholera: studies on reactogenicity and immunogenicity. *J Infect Dis* 1987; 155(1):79-85.

18. Jertborn M, Svennerholm AM, Holmgren J. Evaluation of different immunization schedules for oral cholera B subunit-whole cell vaccine in Swedish volunteers. *Vaccine* 1993;11(10):1007-12.

19. Using a fingerprint recognition system in a vaccine trial to avoid misclassification. *Bull World Health Organ* 2007;85(1):64-7.

20. Bachmann BJ. Pedigrees of some mutant strains of *Escherichia coli* K-12, Bacteriol. Rev. 1972; 36(4): 525-557.

21. US Environmental Protection Agency. *Escherichia coli* K-12 Derivatives Final Risk Assessment: <http://www.epa.gov/biotech_rule/pubs/fra/fra004.htm>, Accessed 27Jan 2010

22. Williams-Smith H. Is it safe to use *Escherichia coli* in recombinant DNA experiments? J. Infectious Diseases, 1978; 137(5): 655-660.

1. United States Food and Drugs Administration Center for Biologics Evaluation and Research. Toxicity Grading Scale for Healthy Adult and Adolescent Volunteers Enrolled in Preventive Vaccine Clinical Trials. available at http://www.fda.gov/cber/gdlns/toxvac.htm, accessed on 4 December 2007.
2. Farrington, Manning. Test statistics and sample size formulae for comparative binomial trials with null hypothesis of non-zero risk difference or non-unity relative risk. Stat Med 1990; 9:1447-54.
