# Supplementary material for: Flexibility of Oral Cholera Vaccine Dosing—A Randomized Controlled Trial Measuring Immune Responses Following Alternative Vaccination Schedules in a Cholera Hyper-Endemic Zone
Source: PLoS Negl Trop Dis. 2015 Mar 12;9(3):e0003574. doi: 10.1371/journal.pntd.0003574 (PMC4357440; doi:10.1371/journal.pntd.0003574)
Supplement: S1 Table — (DOCX) [file pntd.0003574.s003.docx]

### Table S1: Vibriocidal antibody titers and proportion of ≥ 4 fold rise from baseline GMT to *V. cholerae* O1 Inaba (1-5years, 6-17years)

|  | | **1-5 years** | | | **6-17 years** | | |
| --- | --- | --- | --- | --- | --- | --- | --- |
|  |  | **14 day interval  (n=26)** | **28 day interval  (n=25)** | ***p* value** | **14 day interval  (n=58)** | **28 day interval  (n=57)** | ***p* value** |
| **Baseline** | GMT^a^ (95% CI) | 14.1 ( 6.4,31.1) | 69.6 (30.5,159.1) | 0.01 | 80.9 (46.1,142.2) | 98.4 (56.2,172.3) | 0.62 |
| **14 days after  first vaccine dose** | GMT^a^ (95% CI) | 771.3 (300.6, 1979) | 1025 (403.2, 2608) | 0.66 | 1832 ( 1110, 3023) | 2380 ( 1671, 3390) | 0.39 |
|  | GMF^b^ rise | 54.5 (24,123.9) | 14.7 ( 7.5,28.9) | 0.01 | 22.6 (12.3,41.8) | 24.2 (13.9, 42) | 0.87 |
|  | No. who seroconverted^c^ (%) | 25 (96%) | 21 (84%) | 0.19 | 47 (81%) | 52 (91%) | 0.11 |
| **14 days after  second vaccine dose** | GMT^a^ (95% CI) | 490.2 (208.9, 1151) | 695.5 (333.3, 1451) | 0.53 | 1045 (670.2, 1628) | 1093 (744.4, 1604) | 0.88 |
|  | GMF^d^ rise | 34.7 (17.4,68.9) | 9.99 ( 5.5,18.3) | 0.01 | 12.9 ( 7.6, 22) | 11.1 (6.9, 17.7) | 0.67 |
|  | No. who seroconverted^e^ (%) | 24 (92%) | 21 (84%) | 0.42 | 43 (74%) | 42 (74%) | 0.96 |
