# Supplementary material for: Flexibility of Oral Cholera Vaccine Dosing—A Randomized Controlled Trial Measuring Immune Responses Following Alternative Vaccination Schedules in a Cholera Hyper-Endemic Zone
Source: PLoS Negl Trop Dis. 2015 Mar 12;9(3):e0003574. doi: 10.1371/journal.pntd.0003574 (PMC4357440; doi:10.1371/journal.pntd.0003574)
Supplement: S2 Table — (DOCX) [file pntd.0003574.s004.docx]

**Table S2:** Multiple regression analysis of dosing interval effect controlling for baseline GMT

| **Age groups** | **dependent** | **Parameter** | **Estimate** | **p-Value** |
| --- | --- | --- | --- | --- |
| All age (n=336) | Post-dose 2 (log) | 28 day interval group compared to 14 day interval group | -0.13 | 0.33 |
| 1 - 5 years (n=51) | Post-dose 2 (log) | 28 day interval group compared to 14 day interval group | -0.71 | 0.11 |
| 6 - 10 years (n=56) | Post-dose 2 (log) | 28 day interval group compared to 14 day interval group | -0.48 | 0.22 |
| 11 - 17 years (n=59) | Post-dose 2 (log) | 28 day interval group compared to 14 day interval group | 0.42 | 0.22 |
| 18+ years (n=170) | Post-dose 2 (log) | 28 day interval group compared to 14 day interval group | -0.14 | 0.29 |

Multiple linear regression models were fitted to assess dosing interval effect after controlling confounding variable. In the model, the logarithms of vibriocidal titers at post-dose 2 was dependent variable, and the dosing interval status, logarithm of the baseline vibriocidal titers and confounding variable were fitted as the independent variables.
